# Fully-automated segmentation of fluid regions in exudative age-related macular degeneration subjects: Kernel graph cut in neutrosophic domain

**DOI:** 10.1371/journal.pone.0186949

**Published:** 2017-10-23

**Authors:** Abdolreza Rashno, Behzad Nazari, Dara D. Koozekanani, Paul M. Drayna, Saeed Sadri, Hossein Rabbani, Keshab K. Parhi

**Affiliations:** 1 Department of Electrical and Computer Engineering, Isfahan University of Technology, Isfahan, Iran; 2 Department of Electrical and Computer Engineering, University of Minnesota, Minneapolis, MN, United States of America; 3 Department of Ophthalmology and Visual Neurosciences, University of Minnesota, Minneapolis, MN, United States of America; 4 Department of Biomedical Engineering, Medical Image and Signal Processing Research Center, Isfahan University of Medical Sciences, Isfahan, Iran; University of Massachusetts Medical School, UNITED STATES

## Abstract

A *fully-automated* method based on graph shortest path, graph cut and neutrosophic (NS) sets is presented for fluid segmentation in OCT volumes for exudative age related macular degeneration (EAMD) subjects. The proposed method includes three main steps: 1) The inner limiting membrane (ILM) and the retinal pigment epithelium (RPE) layers are segmented using proposed methods based on graph shortest path in NS domain. A *flattened* RPE boundary is calculated such that all three types of fluid regions, intra-retinal, sub-retinal and sub-RPE, are located above it. 2) Seed points for fluid (object) and tissue (background) are initialized for graph cut by the proposed automated method. 3) A *new* cost function is proposed in kernel space, and is minimized with max-flow/min-cut algorithms, leading to a binary segmentation. Important properties of the proposed steps are proven and quantitative performance of each step is analyzed separately. The proposed method is evaluated using a publicly available dataset referred as Optima and a local dataset from the UMN clinic. For fluid segmentation in 2D individual slices, the proposed method outperforms the previously proposed methods by 18%, 21% with respect to the dice coefficient and sensitivity, respectively, on the Optima dataset, and by 16%, 11% and 12% with respect to the dice coefficient, sensitivity and precision, respectively, on the local UMN dataset. Finally, for 3D fluid volume segmentation, the proposed method achieves true positive rate (TPR) and false positive rate (FPR) of 90% and 0.74%, respectively, with a correlation of 95% between automated and expert manual segmentations using linear regression analysis.

## Introduction

**Fig 1:**
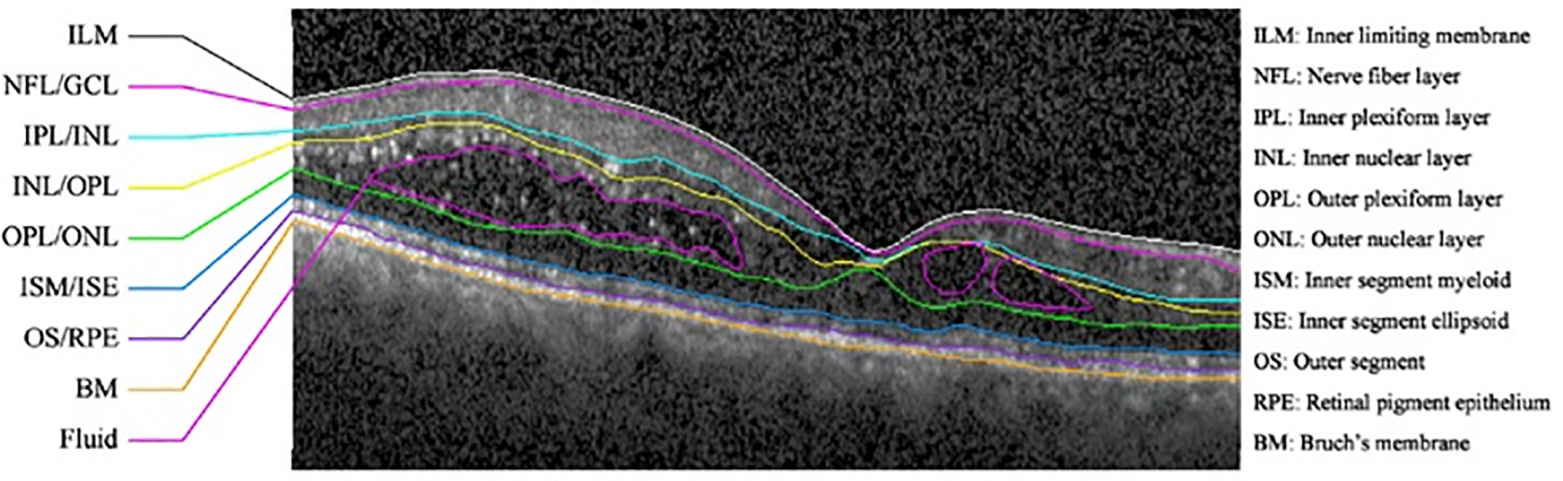
OCT image with layers structure and fluid/cyst regions [17].

Automated OCT images analysis allows detection and quantitative assessment of retinal abnormalities [1, 2]. The analysis of fluid and other abnormalities is a challenging task and has been of greater interest in recent years [3, 4]. In this work, automated analysis of exudative age related macular degeneration (EAMD) is carried out, which is characterized by the growth of abnormal blood vessels from the choroidal vasculature, and the resultant fluid leakage into the intra-retinal, sub-retinal, and sub-retinal pigment epithelium (RPE) spaces. The standard treatment for this condition is guided by the presence and quantity of this fluid [5, 6]. The fluid quantity cannot be routinely measured in clinical practice because commercial algorithms do not directly detect fluid. An OCT image, labeled with the structure of the retinal layers and abnormal fluid/cyst regions, is shown in Fig 1.

Fluid segmentation in OCT images is important for both AMD and diabetic macular edema (DME). With respect to the reported segmentation and quantification of fluid, morphological and pathological features, various aspects have been considered in the literature. These include retinal abnormality type such as AMD/DME, automated/semi-automated (with user interaction), 2D/3D and supervised/not machine learning-based. In [7–11], semi-automated methods were proposed for fluid segmentation in AMD and DME subjects. In [12], a supervised 2D fluid segmentation method was proposed for AMD subjects. In [13–16], supervised 3D fluid segmentation methods were proposed for AMD subjects. In [17–21], supervised 2D fluid segmentation methods were presented for DME subjects. In [22–28], not machine learning-based 2D fluid segmentation methods were proposed for DME subjects. In [29], supervised 3D fluid segmentation method was proposed for DME subjects. Finally, in [30, 31], not machine learning-based 3D fluid segmentation methods were proposed for DME and AMD subjects, respectively. The proposed fluid segmentation method in this paper is a not machine learning-based automated method for EAMD subjects which is applied to 2D slices individually and is used to create a 3D fluid volume for each OCT volume. A preliminary version of this work has been reported in [32].

Neutrosophy is a branch of philosophy which studies the nature and scope of the neutralities and their interactions which is the basis of neutrosophic (NS) logic and NS set [33]. This theory was applied for image segmentation first by Guo *et al* [34] and has subsequently been successfully used for other image processing applications [35–41]. Graph theory is one of the powerful tools for image segmentation due to the benefits of mapping the image pixels (voxels) as well as their relationships onto a graph. Graph theory-based image segmentation makes use of techniques such as minimal spanning tree, graph cut with cost function, graph cut on Markov random field models, shortest path and random walker methods [42]. Graph cut and shortest path methods have been sucessfully applied in image segmentation aplications [43–47].

Fluid segmentation in EAMD subjects is a challenging task due to these reasons: the low contrast of retinal images; variation of size, shape, and location in fluid regions; the similarity between the foreground and background textures; signal intensity changes in subsequent slices in OCT volumes; existence of hard exudates in the form of intensely hyper-reflective structures; and dark regions underneath blood vessels [10, 14]. This paper addresses these challenges and presents approaches to segmentation of fluid regions in OCT volumes of EAMD subjects. The proposed method constitutes three main steps: 1) The inner limiting membrane (ILM) and the retinal pigment epithelium (RPE) layers are segmented using the proposed weight computation equations for graph shortest path in the neutrosophic (NS) domain. Note that in EAMD subjects, ILM segmentation is straightforward while RPE segmentation is a more challenging task due to sub-retinal and sub-RPE fluids which may mislead the graph shortest path method. These challenges are addressed in this paper. Then a *flattened* RPE boundary is calculated such that all types of fluid regions are located above it. It may be noted that the method proposed in [30] uses manual interaction to avoid such challenges. 2) In this work, fluid segmentation is considered as a binary segmentation so that graph cut can be applied. For this task, seed points for fluid (object) and tissue (background) are initialized automatically by the proposed method based on NS theory. 3) To reduce the segmentation errors of the graph cut cost function in [43] at boundaries, a new cost function is proposed in kernel space, and is minimized effectively using min-cut/max-flow algorithms [48]. Finally, several important properties of the proposed steps are presented and then proven. Then quantitative performance of each step is analyzed separately in Section IV.

The rest of this paper is organized as follows. Section II presents a review of the neutrosophic set and graph cut method. Our proposed algorithms are presented in Section III. Experimental results are presented in Sections IV. Finally, conclusion is described in Sections V.

## Review of neutrosophic set and graph cut

### Neutrosophic set and neutrosophic image

Suppose that *A* is a set in neutrosophic domain. Each member *x* in *A* is described by three real subsets named as *T*, *I* and *F* in [0, 1]. Element *x* in *A* is expressed as *x*(*t*, *i*, *f*) which can be interpreted as it is *t*% true, *i*% indeterminacy, and *f*% false [33]. This interpretation depends on the application. In fluid segmentation, our definition is that for each pixel it is *t*% fluid, *f*% non-fluid and the confidence of this assignment is (1 − *i*)%. For using the concept of NS in image processing, an image should be transformed into the neutrosophic domain. The general method for this transformation was proposed by Guo *et al* [34]. For OCT images with layered structures, two transformation methods are proposed here for automated seed initialization and fluid segmentation. These methods will be explained in the next section with details.

### Graph cut

Graph cut is a semi-automated global optimization method for image segmentation. In this method, each pixel in image is represented by a node in the graph, each node (pixel) is linked to its neighboring nodes with the edge cost defined by the difference between pixel gray levels. Also, two additional nodes referred as source and sink are added to represent the foreground and background, respectively. Each node of the graph is connected to source and sink with the edge cost corresponding to the probability that corresponds to how the intensity of that pixel fits into intensity probablities of foreground and background models computed from user seed points. Segmentation problem can be viewed as a binary labeling in graph cut framework which is equivalent to finding the minimum cut in the constructed graph from input image. The energy function *E*(*A*) for image segmentation in graph cut is defined in (1) [43, 49].

$$\begin{array}{c} E(A)=\lambda .R(A)+B(A) \end{array}$$ (1)

$$\begin{array}{c} R\left(A\right)=\sum_{p\in P}^{}R_{p}\left(A_{p}\right) \end{array}$$ (2)

$$\begin{array}{c} B\left(A\right)=\sum_{\{p,q\}\in N}^{}B_{p,q}.\delta \left(A_{p},A_{q}\right) \end{array}$$ (3)

$$\begin{array}{c} \delta \left(A_{p},A_{q}\right)=\{\begin{array}{cc} 1, & \text{if}\;A_{p}=A_{q}\\ 0, & \text{otherwise} \end{array} \end{array}$$ (4)

where λ is a nonnegative parameter that represents a relative importance of the regional property term *R*(*A*) versus the boundary property term *B*(*A*). In regional term *R*(*A*), the individual penalties for assigning pixel *p* to *object* and *background*, *R*_*p*_(*obj*) and *R*_*p*_(*bkg*), respectively, are considered. *A*_*p*_ is the label assigned to pixel *p*. Coefficient *B*_*p*,*q*_ >= 0 is interpreted as a penalty for discontinuity between *p* and *q* [43, 49]. Our proposed algorithm for fluid segmentation in OCT scans is based on graph cut with three improvements which will be discussed in the next section.

## Proposed method

### Transform OCT scans to neutrosophic domain

In [34], it was shown that if the image is transformed to NS domain, the membership set *T* becomes more distinct, which is suitable for segmentation. Also, it was shown that segmentation in NS domain is more robust against noise. In OCT scans with a great deal of noise, it may be a good idea to transform images to NS domain and then apply fluid and layer segmentation methods. This transformation can be useful in OCT image analysis in two main aspects: 1) some abnormalities in OCT scans such as hard exudate regions and hyper-reflective structures affect conventional segmentation methods. These regions can be handled in NS domain to decrease their effects on segmentation. In this research, a new method is proposed to transform OCT scans to NS domain which is consistent with the layered structure of OCT images. In the next step, proposed layer and fluid segmentation methods are applied to images in NS domain. 2) Indeterminacy set in NS domain is used in our proposed cost function for fluid segmentation. As it will be discussed later, our proposed cost function penalizes noisy pixels. Here, pixels with high indeterminacy values are assumed as noisy pixels. The proposed method to transform OCT scans to NS domain is presented in Algorithm 1.

**Algorithm 1** Proposed image transformation to NS domain

1: Inputs: *g* (input image), Output: *T*, *I*.

2: Compute $T\left(i,j\right)=1-\frac{\bar{g(i,j)}-\bar{g_{min}}}{\bar{g_{max}}-\bar{g_{min}}}$.

3: Consider a rectangular Gaussian filter with the dimension of [3, 9].

4: Rotate the filter in 10 different directions to cover 180 degrees of rotation.

5: Apply all filters to image *T* to compute 10 filtered images: *FI*_*k* = 1…10_.

6: Compute *I* as: *I*(*i*, *j*) = *min*_*k*_|*T*(*i*, *j*) − *FI*_*k*_(*i*, *j*)|.

7: Apply λ-correction operation to *T* set as follows:

$$\begin{array}{c} \bar{T}\left(\lambda \right)=\{\begin{array}{cc} T(i,j), & \text{if}\;I(i,j)<\lambda \\ T_{\lambda }^{\prime }\left(i,j\right), & \text{otherwise} \end{array} \end{array}$$ (5)

$$\begin{array}{c} Ind\left(i,j\right)=argmax_{k}\left|T\left(i,j\right)-FI_{k}\left(i,j\right)\right| \end{array}$$ (6)

$$\begin{array}{c} T_{\lambda }^{\prime }\left(i,j\right)=FI_{Ind(i,j)}\left(i,j\right) \end{array}$$ (7)

8: Compute $T=\bar{T}\left(\lambda \right)$.

9: If |*Entropy*(*I*_*t*_) − *Entropy*(*I*_*t*−1_)| < 0.001 go to 10, otherwise go to 5.

10: End.

**Fig 2:**
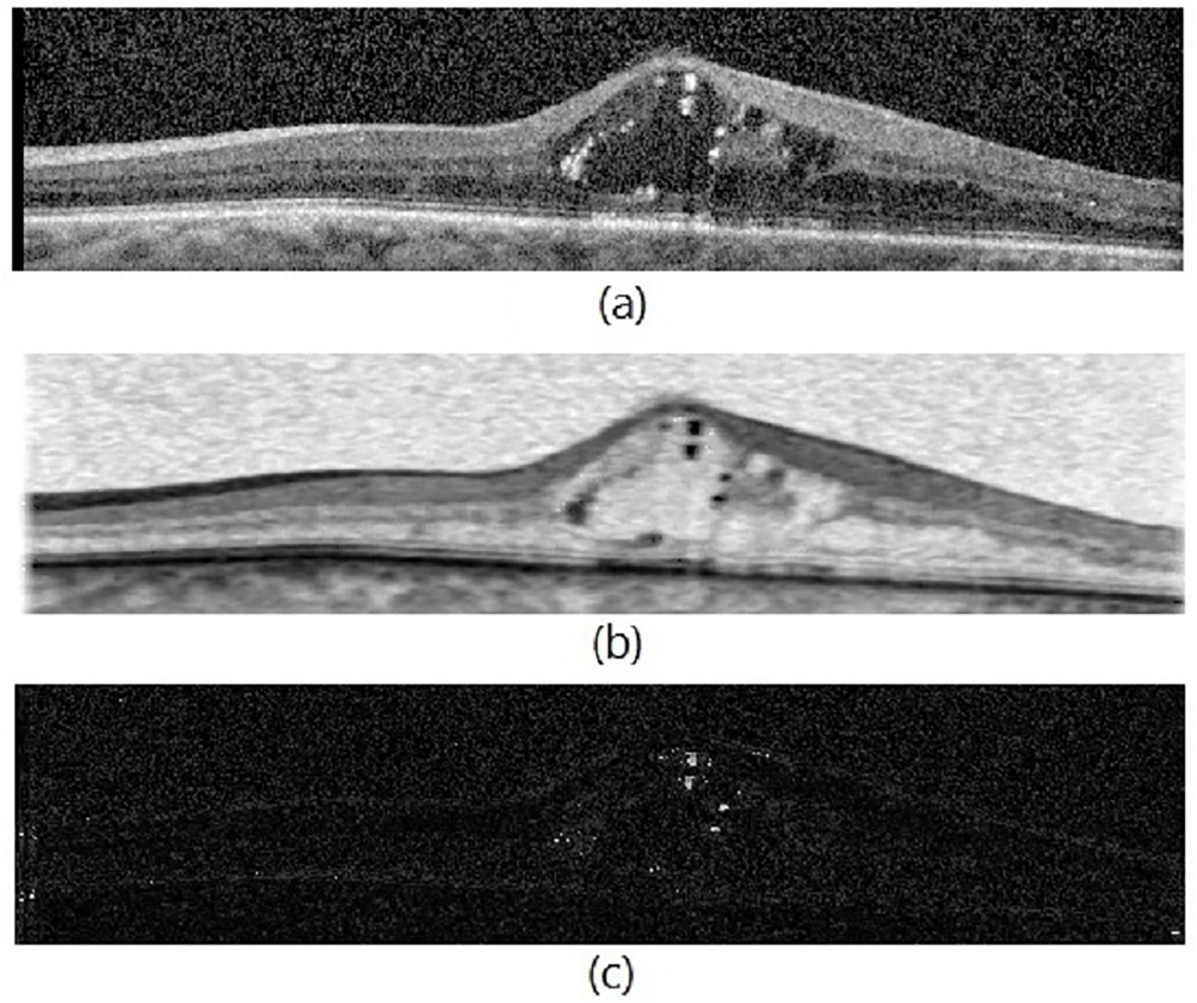
Transformation to NS domain. (a): input OCT scan, (b): subset *T* and (c): subset *I*.

To achieve better segmentation results, several novel approaches are applied to conventional NS transformation which are summarized as follows: 1) Since fluid regions are darker than other regions, the inverse of normalized intensity is considered as *T* set in step 2. Therefore, the proposed method assigns high memberships to pixels in fluid regions and low memberships to pixels in hard exudate regions and hyper-reflective structures such that these regions do not mislead the segmentation methods. Note that proposed layer and fluid segmentation methods are applied to *T* set. 2) A new definition of indeterminacy set is proposed. In indeterminacy definition of conventional NS, the greater the difference between each pixel and the mean of its neighbors in the square window, the greater the indeterminacy. In this definition, higher indeterminacy is assigned to pixels near the OCT layers and the boundaries even though these are not noisy pixels. In the proposed indeterminacy definition (steps 3-6), instead of considering the difference between each pixel and the mean of its surrounding window, the minimum difference between each pixel and the mean of its neighbors in 10 different directions is considered. By this way, for pixels located in layer boundaries, a filter in horizontal direction is selected in step 6 which results in the lowest difference. Thus, the indeterminacy of these pixels is not increased. 3) Instead of *α* − *mean* and *β* − *enhancement* operations in conventional NS, λ-correction operation (λ = 0.7 is chosen based on experiments) is proposed to decrease the noise effect as defined in (5)–(7). In this definition, the very noisy pixels are blurred with the filter which has the greatest difference (the biggest penalty is considered for these pixels). 4) In NS domain, just *T* and *I* sets are used in the subsequent steps of segmentation and *F* is ignored. Fig 2 shows *T* and *I* sets of a transformed OCT scan to NS domain.

### ILM/RPE segmentation

**Fig 3:**
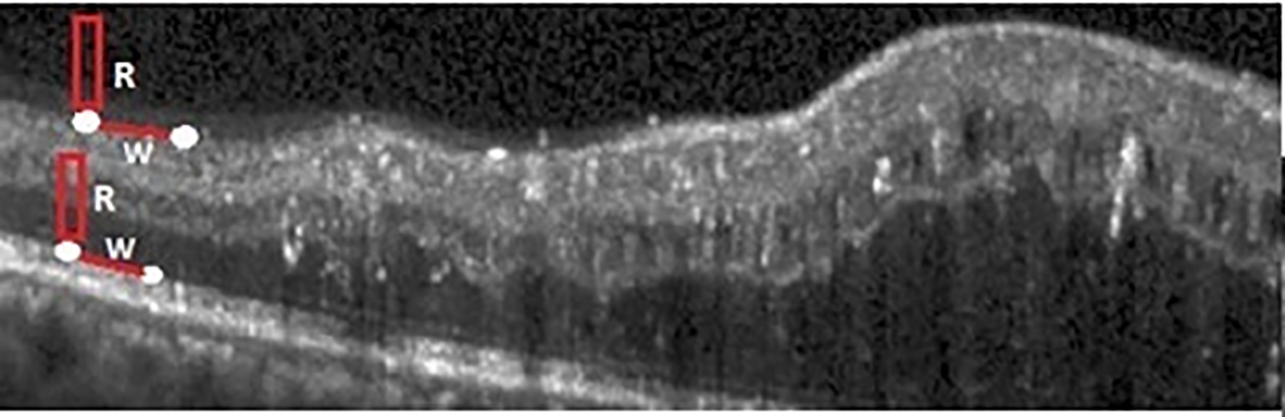
*R* parameter for weight computation in ILM segmentation.

Layer segmentation is not the main contribution of this paper. However, to improve the fluid segmentation results, ILM and RPE, the first and last retinal layers, respectively, are segmented. Since the background regions (the regions above ILM and below RPE) are very similar to fluid regions in both brightness and texture, these regions are ignored after the ILM and RPE layers are segmented. The proposed ILM/RPE segmentation methods for OCT scans of EAMD subjects are derived from the layer segmentation method based on graph shortest path in [46] which was proposed for the OCT layers of normal adult eyes. The general procedure in shortest path method for layer segmentation is that first the graph is constructed from each OCT image by mapping each pixel in the image to one node in a graph. Then, each node is connected to its neighbors with a weight. The main challenge is that edge weights should be calculated such that the edges between pixels located in a layer boundary have the minimum costs and these pixels are good candidates to be selected by the shortest path algorithm. Here, we only consider the local relationship for 8 neighbors of each pixel, so, the 8-regular graph is constructed. The proposed ILM segmentation is as follows: first, the image is filtered with filter *H* for the calculation of vertical gradient of each pixel using (8) and (9).

$$\begin{array}{c} H=[\begin{array}{c} -2\\ 0\\ 2 \end{array}] \end{array}$$ (8)

$$\begin{array}{c} VerGrad=T*H \end{array}$$ (9)

where *T* is the transformed image in NS. This filter is selected based on the fact that ILM pixels are located in the boundary of dark region (background above ILM) and bright region (retina tissue below ILM). The *proposed* weight computation between any two arbitrary pixels (*a*_1_, *b*_1_) and (*a*_2_, *b*_2_) is defined by (10):

$$\begin{array}{c} W\left(\left(a_{1},b_{1}\right),\left(a_{2},b_{2}\right)\right)=4*MaxG-VerGrad\left(a_{1},b_{1}\right)-VerGrad\left(a_{2},b_{2}\right)+2*mean\left(R\right) \end{array}$$ (10)

where *MaxG* is the maximum gray level of the image and *R* is a set of *R* pixels above (*a*_1_, *b*_1_). In this paper *R* is set to 40 based on experiments. Based on the filter *H*, the maximum of *VerGrad* is 2 * *MaxG*. So, the maximum of *VerGrad*(*a*_1_, *b*_1_) + *VerGrad*(*a*_2_, *b*_2_) is 4 * *MaxG*. Pixels which are located in the first layer have the maximum vertical gradient. Therefore, the minimum weight will be assigned between these pixels and they have the highest chance to be selected by the shortest path algorithm. The problem is that in abnormal OCT scans there are pixels in other layers which also have the maximum vertical gradient. Fig 3 shows the pixels which have the maximum vertical gradient (and thus minimum weight) but are not located in the first layer. The weight between these pixels is increased by introducing the term 2 * *mean*(*R*); note that this term is very small for the pixels in ILM. Therefore, it is guaranteed that the pixels in ILM always have the minimum weight.

**Fig 4:**
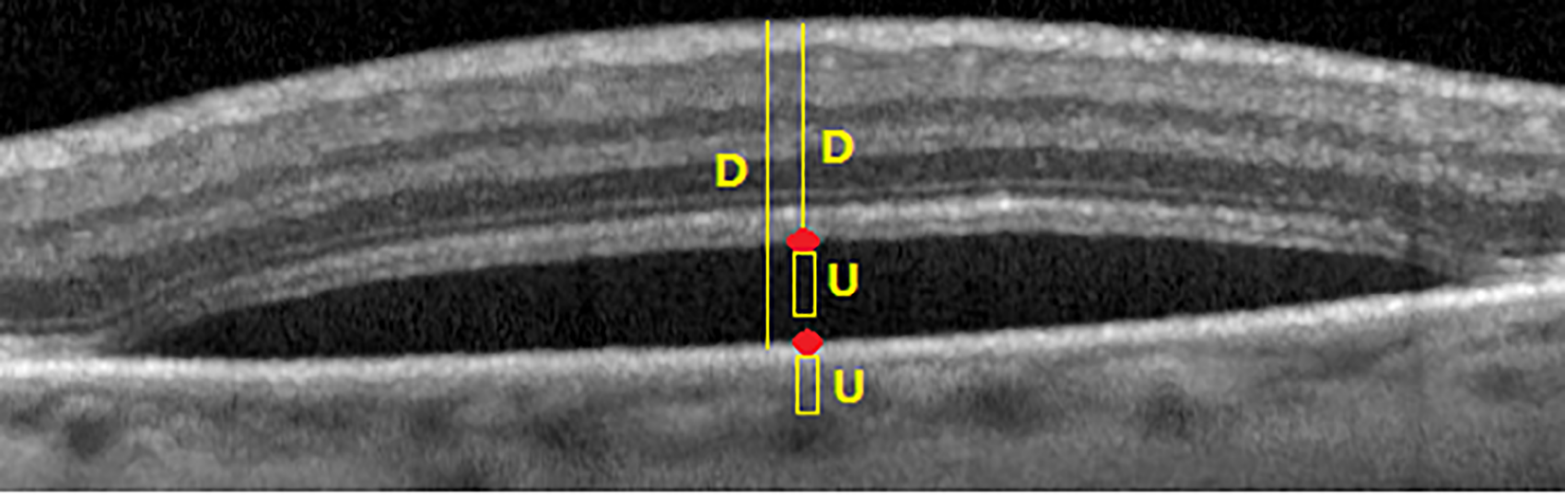
Parameters for weight computation in RPE.

**Fig 5:**
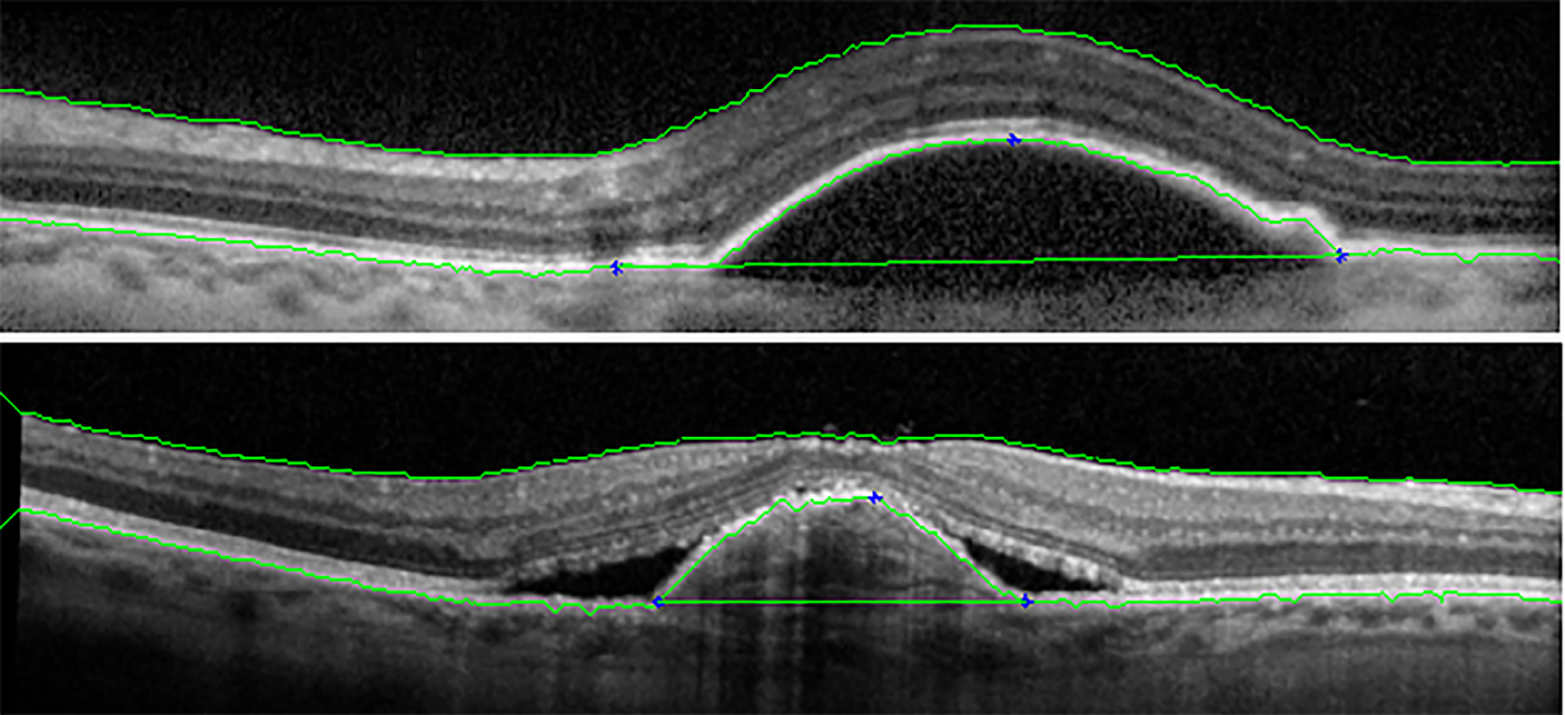
Two samples of ILM and RPE segmentation results.

The procedure for RPE segmentation in EAMD subjects is a more challenging task compared to ILM. In OCT scans of normal eyes, the main feature of RPE pixels is that these pixels are located in the boundary of a bright region (retina tissue above RPE) and a dark region (choroidal vessels below RPE). In normal eyes, if the inverse of filter *H* in ILM segmentation is used, RPE can be segmented. In EAMD subjects, two abnormalities referred as sub-retinal and sub-RPE fluids are the main challenges in RPE segmentation. These challenges are addressed in this paper as follows:

1. For sub-retinal fluid, the proposed weight computation is given by:
  $$W\left(\left(a_{1},b_{1}\right),\left(a_{2},b_{2}\right)\right)=4*MaxG-VerGrad\left(a_{1},b_{1}\right)-VerGrad\left(a_{2},b_{2}\right)-mean\left(U\right)-\beta .D$$ (11)
  where *U* represents a set of *U* pixels below (*a*_1_, *b*_1_) and *D* denotes the vertical distance between (*a*_1_, *b*_1_) and ILM (see Fig 4). In all experiments, *U* and *β* have been set to 10 and 0.004, respectively.
2. For sub-RPE fluid, RPE is elevated by fluid. In these cases, the gradient information is lost and it is not possible to distinguish RPE pixels. Therefore, shortest path method does not work here. For this problem, after RPE segmentation, it is flattened by the proposed Algorithm 2. In this Algorithm first all pixels in RPE are considered as a vector in which *RPE*(*i*) is the height of *ith* pixel in RPE. When the RPE is elevated, a pick (or picks) are created. This pick is determined by steps 3-9 which corresponds to a point with the maximum curvature. Then, the left and right sides where the curve begins and ends are found in steps 11-15 and 17-21, respectively. Finally, left and right sides are connected by 1D linear interpolation which leads to the flattened RPE. Two samples of the layer segmentation results are shown in Fig 5.

The proposed ILM and RPE segmentation guarantees that intra-retinal, sub-retinal and sub-RPE fluids are located between these layers. Therefore, in the proposed fluid segmentation method, just the region between ILM and RPE is considered. In some cases, the layer segmentation errors affect the fluid segmentation algorithm. In Section IV, we will analyze the error of the layer segmentation methods and their effects on fluid segmentation.

**Algorithm 2** RPE Flattening

1: Inputs: All pixels in *RPE*, *W* = 150, *Tr* = 40

2: Output:*Pick*, *PickStart*, *PickEnd*

3: **for** i = 1:length(*RPE*) **do**       ▷ Find a point with the maximum curvature

4:  *L* = 0;*R* = 0;

5:  **for** *j* = 1:*W* **do**

6:   *L* = *L* + *RPE*(*i*) − *RPE*(*i* − *j*);

7:   *R* = *R* + *RPE*(*i* + *j*) − *RPE*(*i*);

8:  *Curvature*(*i*) = *L* + *R*;

9: *Pick* = *max*_*i*_(*Curvature*(*i*))

10: *C* = 1;*Curr* = *Pick*

11: **while** *C* = = 1 **do**         ▷ Find the left side of curvature

12:  **if** *RPE*(*Curr*) < *RPE*(*Curr* − *Tr*) **then**

13:   *Curr* = *Curr* − 1;

14:  **else** *C* = 0;

15:  *PickStart* = *Curr*;

16: *C* = 1;*Curr* = *Pick*

17: **while** *C* = = 1 **do**         ▷ Find the right side of curvature

18:  **if** *RPE*(*Curr*) < *RPE*(*Curr* + *Tr*) **then**

19:   *Curr* = *Curr* + 1;

20:  **else** *C* = 0;

21:  *PickEnd* = *Curr*;

22: Connect PickStart to PickEnd using 1D linear interpolation

23: End.

### Fluid segmentation

In this paper, graph cut is developed for fluid segmentation. In this Section, following cases are explained: 1) Why the proposed fluid segmentation method is based on graph cut, 2) what are the limitations of graph cut for this application, and 3) how we address these limitations.

#### Why graph cut

Optimization based on variational methods relies on approximating numerical approaches that must be very carefully designed to achieve the robustness. Also, convergence of such methods is a non-trivial issue. In contrast, graph cut image segmentation relies on powerful combinatorial optimization methods which are very straightforward, robust and repeatable numerically [43]. Also, in [50], the practical efficiency of combinatorial min-cut/maxflow algorithms for solving optimization problem in graph cut problem was studied. Finally, region, boundary, and shape priors can be integrated in the cost functions which are optimized in graph cut [43, 50].

#### Graph cut limitations in fluid segmentation

**Fig 6:**
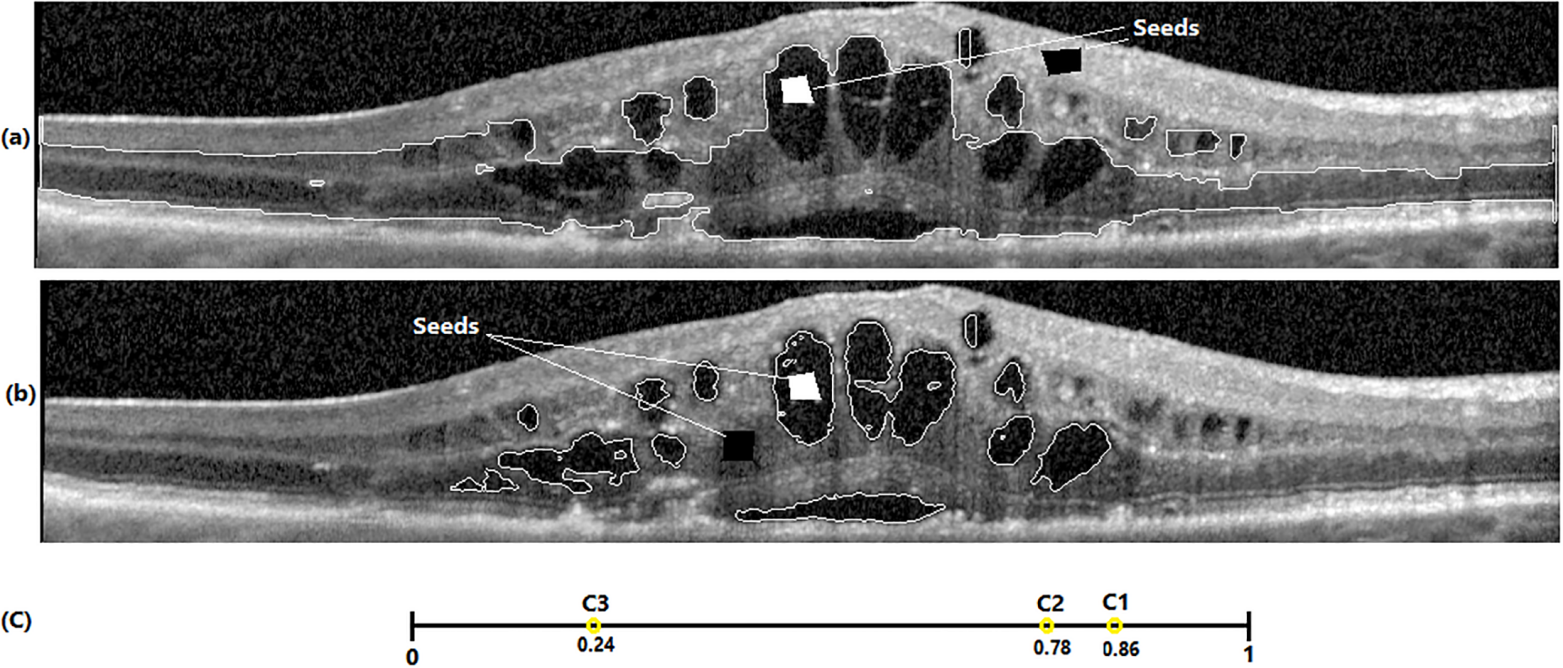
Effect of seed initialization in fluid segmentation. (a) and (b): two different seed point sets (c): center of fluid seeds (C1), tissue seeds in (a) (C3) and (b) (C2).

Beside the computational benefits of graph cut, there are three limitations in graph cut in our problem of interest, i.e., fluid segmentation: 1) Basically, graph cut is a semi-automated method and needs user seed points for regional term and hard constraints while we focus on developing a fully-automated method for fluid segmentation. 2) Graph cut is well-developed for the optimization of binary cost functions and consequently binary image segmentation while in OCT scans there are at least 13 different texture structures in retinal layers. 3) Graph cut is very sensitive to seed points. Fig 6(a) and 6(b) show the fluid segmentations for two different seed points which demonstrate the effect of seed points in fluid segmentation results. These limitations are addressed as follows:

#### Automated seed initialization for graph cut

**Fig 7:**
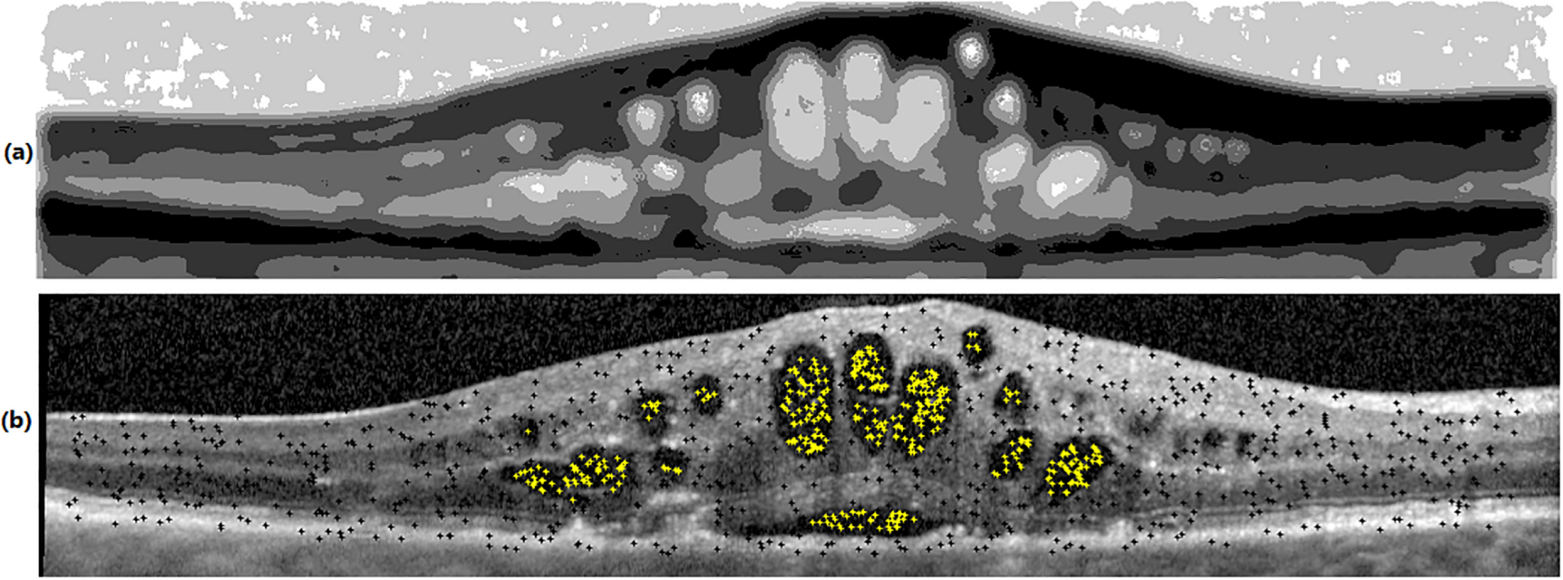
Seed initialization in fluid segmentation. (a) coarse partitions in NS domain (b) final seed initialization result.

In this research, an automated method (without needing user interaction) is proposed for seed initialization in Algorithm 3. It considers the fluid regions as object and tissue regions as background. Automated seed points obtained from Algorithm 3 are depicted in Fig 7(b) in which fluid and tissue seed points are illustrated in white and black colors, respectively.

**Algorithm 3** Automated seed initialization in NS domain.

1: Inputs: *g* (input image), Number of fluid pixels: *FN* (= 500) and number of tissue pixels: *TN* (= 700)

2: Output: seed point sets *Fl*, *Ti*.

3: Compute:

4: $T\left(i,j\right)=1-\frac{\bar{g(i,j)}-\bar{g_{min}}}{\bar{g_{max}}-\bar{g_{min}}}$, $I\left(i,j\right)=\frac{\delta \left(i,j\right)-\delta_{min}}{\delta_{max}-\delta_{min}}$, $\delta (i,j)=|g\left(i,j\right)-\bar{g(i,j)}|$.

5: Apply the *α* − *mean* and *β* − *enhancement* operations proposed in [34] to subset *T*.

6: If |*Entropy*(*I*_*i*_) − *Entropy*(*I*_*i*−1_)| < 0.001 go to 7, otherwise go to 4.

7: Apply *k* − *means* clustering on the subset *T* to have a coarse partitioning (see Fig 7(a)).

8: Sort clusters from bigger to smaller cluster centers.

9: Fluid seed points: select *FN* points randomly from the first two clusters and save them in *Fl* set (see section “Clusters for fluid and tissue seed sets”).

10: Tissue seed points: from remaining (*k* − 2) clusters, select (*Np*_*i*_ × *TN*) pixels from cluster *i*(*i* = 3…*k*) randomly and save them in Ti set: $p_{i}=\frac{1}{2^{i-2}}$, $Np_{i}=\frac{p_{i}}{\sum_{i=3}^{k}p_{i}}$

11: Return *Fl* and *Ti* sets.

#### Cost function design for graph cut

Obtained seed points from Algorithm 3 are presented for graph cut in [43] for fluid segmentation. As it will be shown visually and quantitatively in section IV, fluid regions close to the boundaries are lost in segmentation. For increasing segmentation accuracy, a new cost function based on kernel mapping is designed for graph cut. In kernel mapping, data is transformed to the higher dimensional space which can be expressed with a kernel function without explicit evaluation of the transform [51]. Kernel concept was first integrated with graph cut by Salah *et al.* for automated multi-region image segmentation [44]. Proposed method in [44] segments images to *k* regions without user interaction. The main problem lies in determining what *k* value is appropriate for each OCT scan. One of the main advantage of the proposed seed initialization method is that it does not need the number of clusters (segments) since it is a binary segmentation which considers fluid regions as object and non-fluid regions as background. Therefore, the cost function proposed in [44] cannot be used here. In this work, a new kernel-based cost function is proposed in (12) for binary segmentation which uses seed points obtained from Algorithm 3.

$$\begin{array}{c} E\left(A\right)=\lambda_{1}.\;\sum_{p\in P}^{}R_{p}\left(A_{p}\right)+\lambda_{2}.\;\sum_{\{p,q\}\in N}^{}B_{p,q}.\delta \left(A_{p},A_{q}\right) \end{array}$$ (12)

$$\begin{array}{c} \begin{array}{c} R1_{p}\left(A_{p}\right)={\left(\phi \left(\mu_{A_{p}}\right)-\phi \left(p\right)\right)}^{2}={\left(\phi \left(\mu_{A_{p}}\right)-\phi \left(p\right)\right)}^{T}.\left(\phi \left(\mu_{A_{p}}\right)-\phi \left(p\right)\right)\\ =\phi {\left(\mu_{A_{p}}\right)}^{T}.\phi \left(\mu_{A_{p}}\right)+\phi {\left(\mu_{p}\right)}^{T}.\phi \left(\mu_{p}\right)-2\phi {\left(\mu_{A_{p}}\right)}^{T}.\phi \left(\mu_{p}\right)\\ =K\left(\mu_{A_{p}},\mu_{A_{p}}\right)+K\left(p,p\right)-2K\left(\mu_{A_{p}},p\right)=2-2.exp\left(-\left({\left(\mu_{A_{p}}-p\right)}^{2}\right)/\left(2.\sigma^{2}\right)\right) \end{array} \end{array}$$ (13)

$$\begin{array}{c} \begin{array}{c} R_{p}{(}^{\prime }O^{\prime })={\left(\left(1-I_{p}\right).R1_{p}{(}^{\prime }O^{\prime })\right)}^{GE(R1_{p}{(}^{\prime }O^{\prime }),R1_{p}{(}^{\prime }B^{\prime }))}\\ \times {R1_{p}{(}^{\prime }O^{\prime })}^{GE(R1_{p}{(}^{\prime }B^{\prime }),R1_{p}{(}^{\prime }O^{\prime }))} \end{array} \end{array}$$ (14)

$$\begin{array}{c} \begin{array}{c} R_{p}{(}^{\prime }B^{\prime })={\left(\left(1-I_{p}\right).R1_{p}{(}^{\prime }B^{\prime })\right)}^{GE(R1_{p}{(}^{\prime }B^{\prime }),R1_{p}{(}^{\prime }O^{\prime }))}\\ \times {R1_{p}{(}^{\prime }B^{\prime })}^{GE(R1_{p}{(}^{\prime }O^{\prime }),R1_{p}{(}^{\prime }B^{\prime }))} \end{array} \end{array}$$ (15)

$$\begin{array}{c} GE\left(a,b\right)=\{\begin{array}{cc} 1: & a>b\\ 0: & \text{otherwise} \end{array} \end{array}$$ (16)

where *A*_*p*_ = {′*O*′,′ *B*′} represents the label of pixel *p* which is either object (fluid) or background (tissue) and $\mu_{A_{p}}$ is the average of seed points with the label of *A*_*p*_. λ_1_ and λ_2_ are the weights for region and boundary terms, respectively. *ϕ* maps pixel intensities to higher dimension. For this cost function parameters are set with λ_1_ = 10^12^, λ_2_ = 10^8^ and *σ* = 0.1. Based on Mercer’’s theorem, any continuous, symmetric and positive semidefinite kernel function can be expressed as a dot product in a high-dimensional space, without any need to know explicitly the mapping [44, 51]. In (13), centers of the object and background seeds (obtained from Algorithm 3) are used and then the RBF kernel is used since it has the best accuracy in fluid segmentation. Finally, the boundary term in (12) is same as the boundary term proposed in [43].

#### Final fluid segmentation algorithm

Final fluid segmentation scheme is described in Algorithm 4.

**Algorithm 4** Proposed fluid segmentation method.

1: Transform the input OCT scan to *T* and *I* sets in NS domain with Algorithm 1.

2: Segment ILM and OPL layers of *T*.

3: Initialize seed points for fluid and tissue with Algorithm 3.

4: Construct a graph for the proposed cost function with the same way proposed in [43].

5: Minimize (12) by finding minimum cut of the constructed graph in 4 with min-cut/max-flow algorithms in [48].

6: Ignore segmented regions above ILM and below RPE and regions under *Tr* pixels.

7: Return all segmented regions as fluid (object) in graph cut segmentation.

The proposed methods satisfy certain properties. These are stated and proven below.

***Property 1***. Proposed weight computation in (11) does not mislead the shortest path methods in EAMD subjects with sub-retinal fluid.

**Fig 8:**
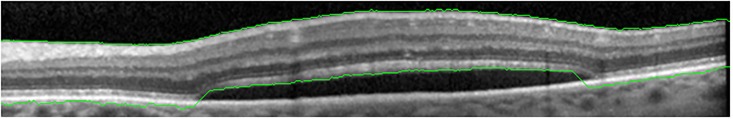
Sub-retinal fluid misleads shortest path algorithm.

**Fig 9:**
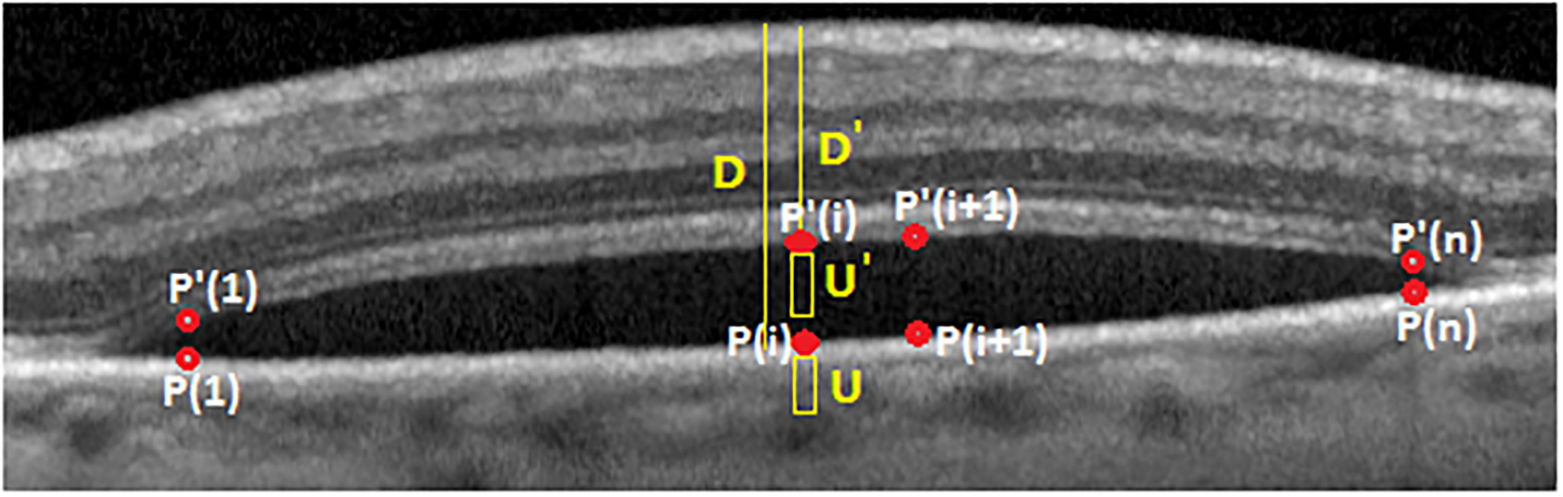
RPE weight computation.

*Proof.* In sub-retinal fluid, there are pixels above fluid regions with high vertical gradients (similar to RPE pixels) which mislead the shortest path algorithm. Therefore, shortest path algorithm follows a path above sub-retinal fluid instead of RPE (see Fig 8). Suppose that there are *n* pixels which mislead the shortest path method. These pixels are depicted in Fig 9 with the set {*P*′(1), …, *P*′(*i*), *P*′(*i* + 1), …, *P*′(*n*)}. These pixels are referred as “above RPE” pixels. There is another set {*P*(1), …, *P*(*i*), *P*(*i* + 1), …, *P*(*n*)} referred as “under RPE” pixels which should be selected by shortest path method. Note that corresponding pixels in these sets have the same column number. Each pixel *P*(*i*) has a column and row number of *a*_*i*_ and *b*_*i*_, respectively. Therefore, it is represented by (*a*_*i*_, *b*_*i*_). What we are showing here is how the proposed weight computation leads the shortest path algorithm to select “under RPE” pixel set instead of “above RPE”.

In (11), for *D*, *D*′, *U* and *U*′ parameters it is clear that:

$$\begin{array}{c} D^{\prime }<D \end{array}$$ (17)

$$\begin{array}{c} U^{\prime }<U \end{array}$$ (18)

The term 4 * *MaxG* is same for both sets. To evaluate the relation between *VerGrad* terms two cases are possible:

1. $VerGrad\left(a_{i}^{\prime },b_{i}^{\prime }\right)<=VerGrad\left(a_{i},b_{i}\right)$ and $VerGrad\left(a_{i+1}^{\prime },b_{i+1}^{\prime }\right)<=VerGrad\left(a_{i+1},b_{i+1}\right)$
  In this case:
  $$\begin{array}{c} \begin{array}{c} VerGrad\left(a_{i}^{\prime },b_{i}^{\prime }\right)+VerGrad\left(a_{i+1}^{\prime },b_{i+1}^{\prime }\right)+mean\left(U^{\prime }\right)+\beta .D^{\prime }<VerGrad\left(a_{i},b_{i}\right)\\ +VerGrad\left(a_{i+1},b_{i+1}\right)+mean\left(U\right)+\beta .D \end{array} \end{array}$$ (19)
  Therefore:
  $$\begin{array}{c} W\left(\left(a_{i},b_{i}\right),\left(a_{i+1},b_{i+1}\right)\right)<W\left(\left(a_{1}^{\prime },b_{i}^{\prime }\right),\left(a_{i+1}^{\prime },b_{i+1}^{\prime }\right)\right) \end{array}$$ (20)
2. $VerGrad\left(a_{i}^{\prime },b_{i}^{\prime }\right)>VerGrad\left(a_{i},b_{i}\right)$ and $VerGrad\left(a_{i+1}^{\prime },b_{i+1}^{\prime }\right)>VerGrad\left(a_{i+1},b_{i+1}\right)$
  In this case:
  $$\begin{array}{c} mean\left(U\right)-mean\left(U^{\prime }\right)>VerGrad\left(a_{i+1}^{\prime },b_{i+1}^{\prime }\right)-VerGrad\left(a_{i+1},b_{i+1}\right) \end{array}$$ (21)
  $$\begin{array}{c} D-D^{\prime }>VerGrad\left(a_{i}^{\prime },b_{i}^{\prime }\right)-VerGrad\left(a_{i},b_{i}\right) \end{array}$$ (22)
  It may be mentioned that *U* is a set of pixels in choroid region while *U*′ is in fluid region. Therefore, inequality (21) is almost true. In inequality (22), the left side is based on distance while the right side is based on gradient. To have a bigger quantity in the left side and also have consistency in both sides, a *β* parameter is multiplied to the left side. Therefore, it can be concluded that:
  $$\begin{array}{c} \begin{array}{c} \beta \left(D-D^{\prime }\right)+\left(mean\left(U\right)-mean\left(U^{\prime }\right)\right)>VerGrad\left(a_{i}^{\prime },b_{i}^{\prime }\right)-VerGrad\left(a_{i},b_{i}\right)+\\ VerGrad\left(a_{i+1}^{\prime },b_{i+1}^{\prime }\right)-VerGrad(a_{i+1},b_{i+1}) \end{array} \end{array}$$ (23)
  Therefore,
  $$\begin{array}{c} \begin{array}{c} VerGrad\left(a_{i}^{\prime },b_{i}^{\prime }\right)+VerGrad\left(a_{i+1}^{\prime },b_{i+1}^{\prime }\right)+mean\left(U^{\prime }\right)+\beta .D^{\prime }<VerGrad\left(a_{i},b_{i}\right)+\\ VerGrad\left(a_{i+1},b_{i+1}\right)+mean\left(U\right)+\beta .D \end{array} \end{array}$$ (24)
  which leads to:
  $$\begin{array}{c} W\left(\left(a_{i},b_{i}\right),\left(a_{i+1},b_{i+1}\right)\right)<W\left(\left(a_{1}^{\prime },b_{i}^{\prime }\right),\left(a_{i+1}^{\prime },b_{i+1}^{\prime }\right)\right) \end{array}$$ (25)

From (20)–(25), we see that the proposed weight computation in (11) assigns smaller weights to “under RPE” pixels ({*P*(1), …, *P*(*i*), *P*(*i* + 1), …, *P*(*n*)}). Therefore, shortest path method does not mislead for “above RPE” pixels.

It should be noted that although inequality (23) is almost true, it does not hold for all cases. It leads to error in RPE segmentation. This margin error is reported in Table 1.

**Table 1:** ILM/RPE segmentation errors.

	Num. of scans	ILM errors	RPE errors
UMN dataset	600	1	34
Optima dataset	196	0	6

***Property 2***. Initialized seeds by Algorithm 3 lead to more accurate results in fluid segmentation in graph cut.

*Proof*. In Algorithm 3, OCT images are first transformed to NS domain in steps 3-6. In this transformation, high memberships are assigned to fluid pixels and then noisy pixels are penalized with an averaging filter of dimension 11 × 11. This filter is used in *α* − *mean* and *β* − *enhancement* operations. After that, *k* − *means* clustering is applied which leads to a coarse partitioned image with *k* clusters (Fig 7(a)). Based on the examples in Fig 6(a) and 6(b) with the same seed points for fluid, it is clear that when the seed points for tissue are selected from bright regions with the average of C3 = 0.24 shown in Fig 6(c), too many false positve pixels are assigned as fluid. This is because many dark and non-fluid pixels are far from tissue seeds. Therefore, these pixels are assigned as fluid. In contrast, if the tissue seed points are selected from darker regions (C2 = 0.78), graph cut assigns all dark and non-fluid pixels to tissue (because these pixels are close to tissue seed points) and consequently, the number of false positive pixels is decreased significantly. In Algorithm 3, fluid seed points are selected from two clusters with the highest cluster centers, and tissue seed points are selected from remaining (*k* − 2) clusters. Based on step 10 of Algorithm 3, $\frac{1}{2^{i-2}}$ percent of tissue seed points are selected from cluster *i*(*i* = 3, 4, 5, …, (*k* − 2)). Therefore,

$$\begin{array}{c} \frac{1}{2^{i-2}}=\frac{1}{2^{i}}\left(2\right)+\frac{1}{2^{i}}\left(1\right)+\frac{1}{2^{i}}\left(1\right)>\frac{1}{2^{i}}\left(2\right)+\frac{1}{2^{i}}\left(1\right)+\frac{1}{2^{i}}\left(1\right)-{\left(\frac{1}{2}\right)}^{k-2i-2} \end{array}$$ (26)

$$\begin{array}{c} \frac{1}{2^{i-2}}>\frac{1}{2^{i}}\left(2\right)+\frac{1}{2^{i}}\left(1\right)+\frac{1}{2^{i}}\left(\frac{\frac{1}{2}-(\frac{1}{2}{)}^{k-i-1}}{\frac{1}{2}}\right) \end{array}$$ (27)

$$\begin{array}{c} \left(\frac{\frac{1}{2}-(\frac{1}{2}{)}^{k-i-1}}{\frac{1}{2}}\right)={\frac{1}{2}}^{1}+{\frac{1}{2}}^{2}+\ldots +{\left(\frac{1}{2}\right)}^{k-i-2} \end{array}$$ (28)

By substituting (28) in (27):

$$\begin{array}{c} \frac{1}{2^{i-2}}>\frac{1}{2^{i-1}}+\frac{1}{2^{i}}+\frac{1}{2^{i+1}}+\ldots +\frac{1}{2^{k-2}} \end{array}$$ (29)

Therefore,

$$\begin{array}{c} p_{i}>p_{i+1}+p_{i+2}+p_{i+3}+\ldots +p_{k} \end{array}$$ (30)

And consequently,

$$\begin{array}{c} Np_{i}>\left(Np_{i+1}+Np_{i+2}+Np_{i+3}+\ldots +Np_{k}\right) \end{array}$$ (31)

It is proven that $Np_{i}>\sum_{h=i+1}^{k}Np_{h}$ which means that more seed points are selected from clusters which are closer to fluid clusters. As shown in Fig 6, the proposed approach decreases the false positive pixels significantly in fluid segmentation.

In Section IV, the robustness of the proposed method in Algorithm 3 will be shown quantitatively.

***Property 3***. Proposed cost function for graph cut penalizes the high indeterminacy pixels to decrease the effect of these pixels in segmentation.

*Proof*. The effect of noise is considered with indeterminacy set *I* in NS domain in Algorithm 1. Based on the graph construction procedure in [43] for regional term, *R*_*p*_(′*B*′) is the weight between pixels and Object terminal node. In (13), centers of the fluid and tissue seeds (obtained from Algorithm 3) are used for computing the regional term. It is clear that pixels which are likely fluid are strongly connected to Object terminal node with large weights (*R*_*p*_(′*B*′)). If these pixels are noisy (with low values in (1 − *I*)), their strong connection will be weaker by multiplier (1 − *I*) in (32). This procedure is applied in an inverse manner for noisy non-fluid pixels in (33)

$$\begin{array}{c} R_{p}{(}^{\prime }B^{\prime })=\{\begin{array}{cc} \left(1-I_{p}\right).R1_{p}{(}^{\prime }B^{\prime }), & :R1_{p}{(}^{\prime }B^{\prime })>R1_{p}{(}^{\prime }O^{\prime })\\ R1_{p}{(}^{\prime }B^{\prime }), & \text{otherwise} \end{array} \end{array}$$ (32)

$$\begin{array}{c} R_{p}{(}^{\prime }O^{\prime })=\{\begin{array}{cc} \left(1-I_{p}\right).R1_{p}{(}^{\prime }O^{\prime }), & :R1_{p}{(}^{\prime }O^{\prime })>R1_{p}{(}^{\prime }B^{\prime })\\ R1_{p}{(}^{\prime }O^{\prime }), & \text{otherwise} \end{array} \end{array}$$ (33)

To show the effectiveness of the proposed cost function, the segmentation results of this cost function are compared with the cost functions in [43, 44] qualitatively and quantitatively in Section IV.

## Experimental results

### Datasets

In this work, two datasets have been used for the evaluation of the proposed fluid segmentation method. The first dataset is a local dataset from the UMN ophthalmology clinic containing 600 OCT scans collected from 24 EAMD subjects which were taken using the Heidelberg Spectralis imaging system. We have selected 25 scans with the highest area of fluid among about 100 scans in each subject. Scans are obtained by averaging 12-19 frames with the resolution of 5.88*μ*m/pixel along the length and 3.87*μ*m/pixel along the width. Fluid regions were segmented by two UMN ophthalmologists (DDK and PMD). The experimental procedures involving human subjects described in this paper were approved by the Institutional Review Board (IRB) at the University of Minnesota. The second dataset is from the OPTIMA Cyst Segmentation Challenge and contains 4 subjects with 49 images per subject where the image resolution varies from 512x496 to 512x1024. This dataset can be found at: http://optima.meduniwien.ac.at/challenges/optima-segmentation-challenge-1/.

### ILM/RPE segmentation errors

ILM/RPE segmentation separates retinal tissue from background. After applying fluid segmentation method, just the segmented regions between ILM and RPE are considered. Therefore, if ILM and RPE are segmented below and above fluid regions, respectively, these regions will be lost and these cases are considered as layer segmentation errors which are reported in Table 1. It can be concluded that there are more errors in RPE rather than ILM which stems from RPE elevation and sub-retinal fluid in EAMD subjects. Also, RPE errors in UMN dataset are much more than Optima dataset. UMN dataset is collected from EAMD subjects and there are great deal of sub-RPE and sub-retinal fluid regions which adversely affect proposed RPE segmentation algorithm while Optima is a dataset for early AMD subjects with less sub-RPE fluid regions.

### 2D fluid segmentation results

The 2D fluid segmentation results of the proposed method are compared with the ground truth annotated by two experts, graph cut (GC) [43] and kernel graph cut (KGC) [44]. For the Optima dataset, we further compare our results with those of [31, 52, 53] which correspond to the top three ranked solutions in this challenge.

**Fig 10:**
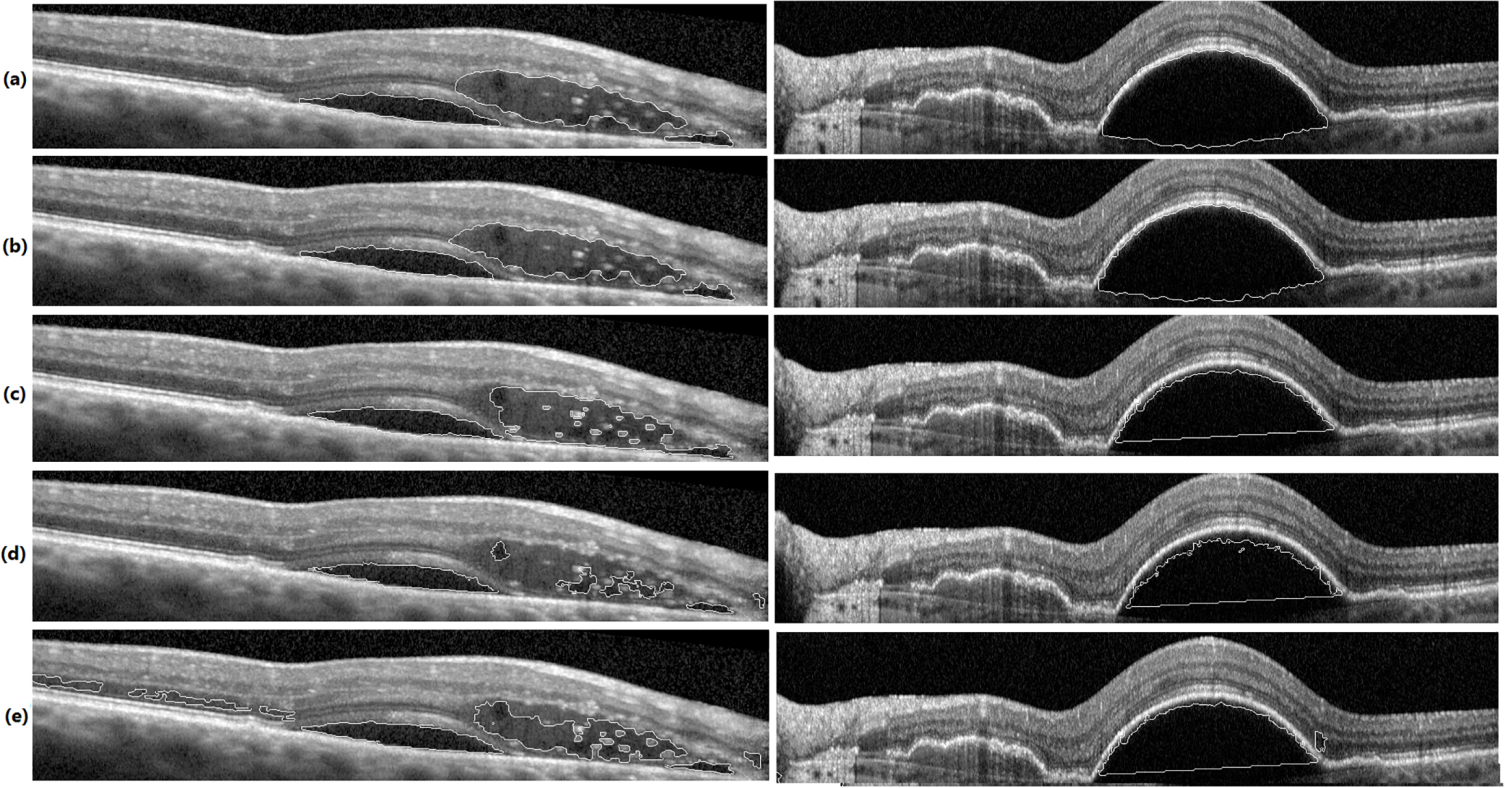
Final fluid segmentation results in UMN dataset. Segmentation by: (a): expert 1, (b): expert 2, (c): proposed method, (d): GC method and (e): KGC method.

**Fig 11:**
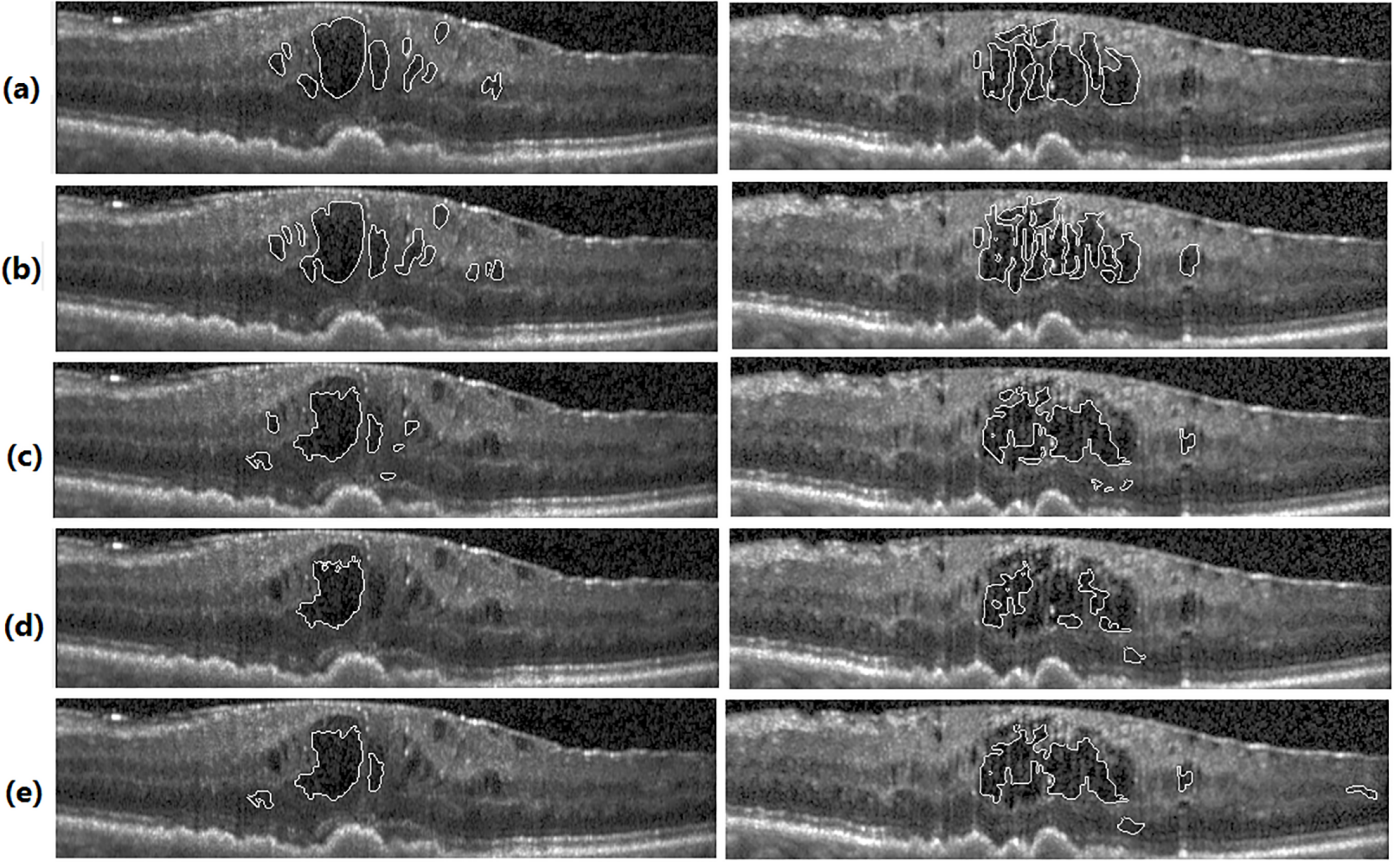
Final fluid segmentation results in Optima dataset. Segmentation by: (a): expert 1, (b): expert 2, (c): proposed method, (d): GC method and (e): KGC method.

**Fig 12:**
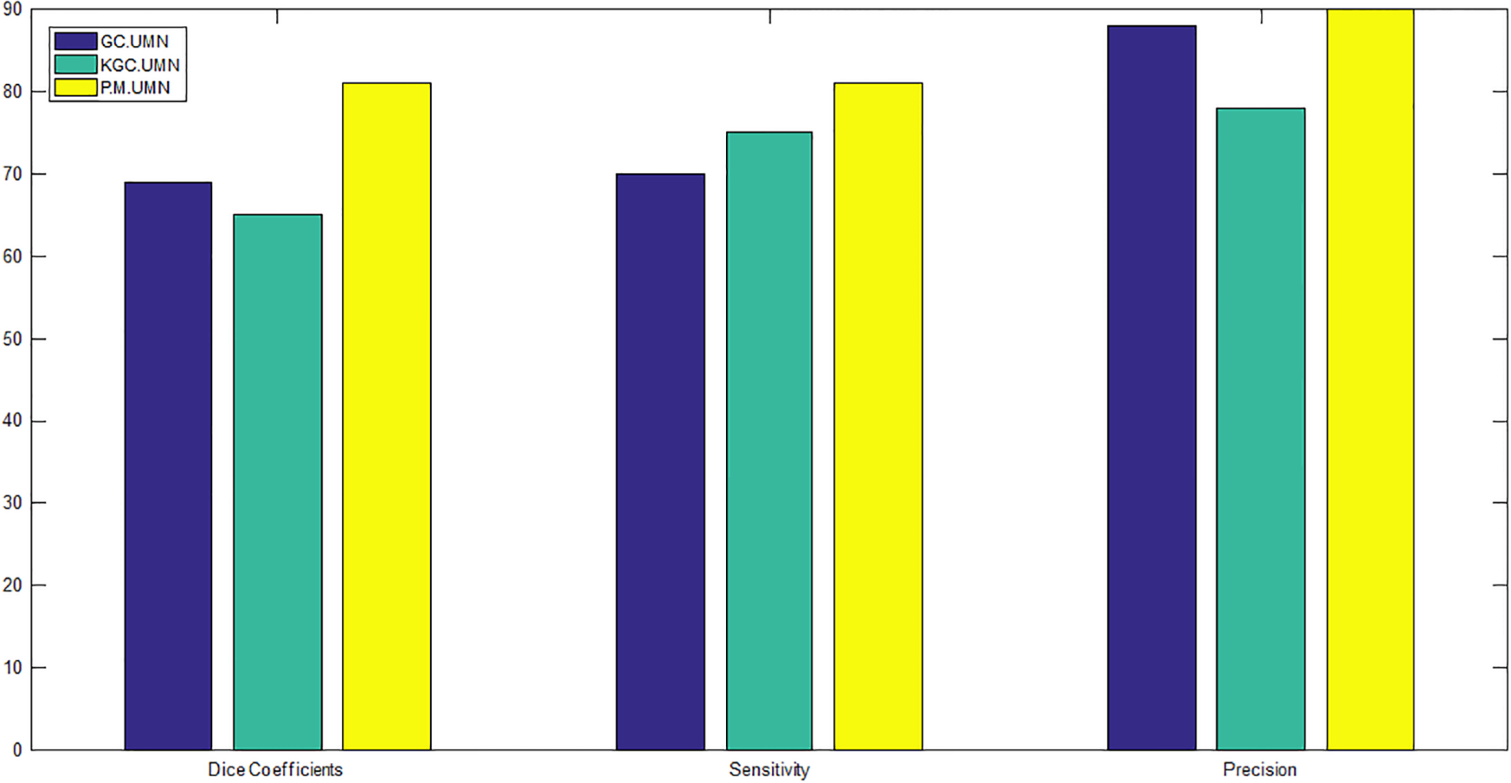
Average dice coefficients, sensitivity and precision of all subjects in UMN datasets.

**Fig 13:**
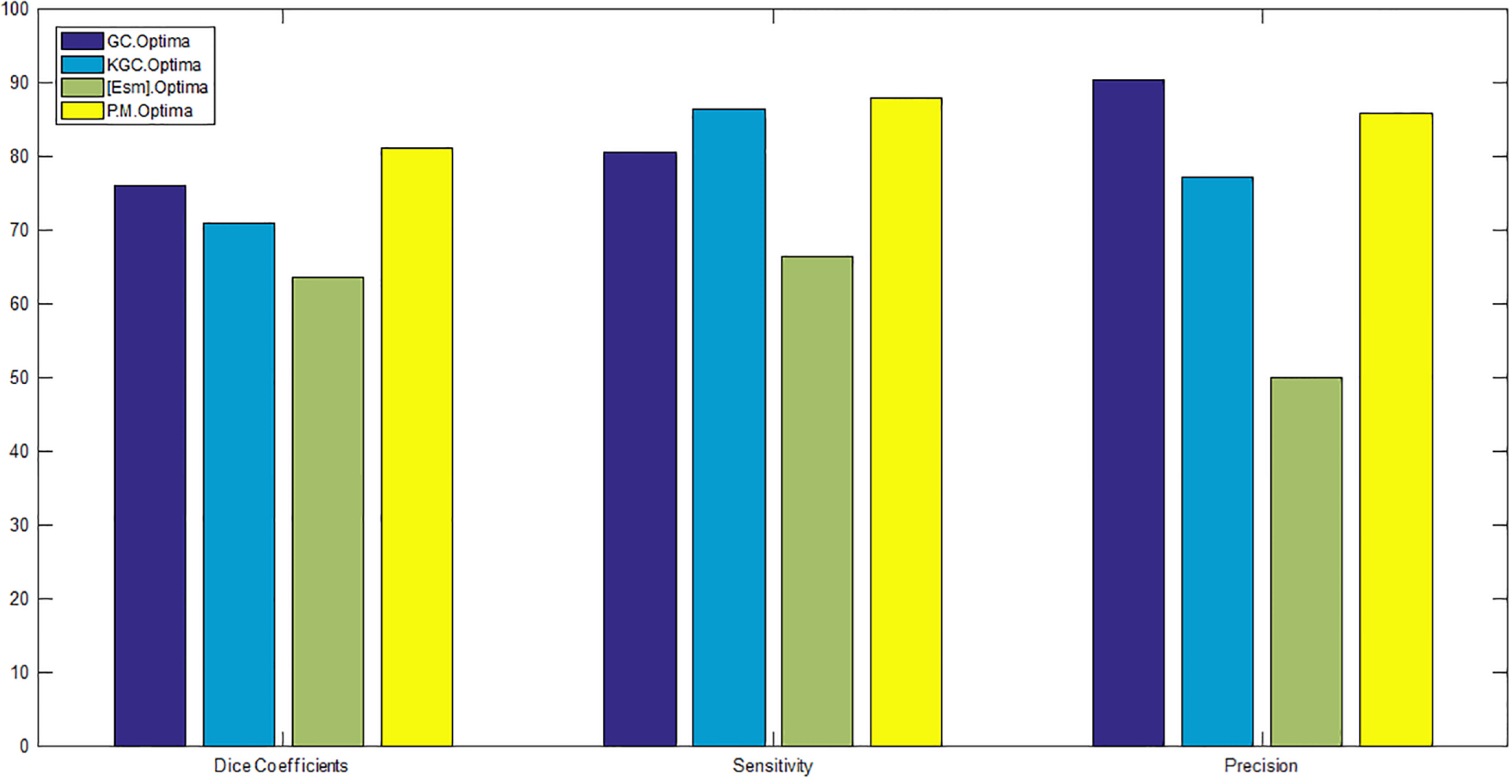
Average dice coefficients, sensitivity and precision of all subjects in Optima datasets.

For having a fair comparison, in both GC and KGC, segmented ILM and RPE layers are used in post processing. Also, in GC, the automated seed points obtained from Algorithm 3 are used since GC is basically an interactive method. Segmentation results in UMN and Optima datasets are depicted in Figs 10 and 11, respectively. From these figures, it is clear that the proposed method segments boundaries more accurately than GC since it performs segmentation in higher kernel space. Although KGC has good results at the boundaries, appropriate number of clusters (*NC*) in this method should be assigned accurately. Here, NC = 7 is considered which leads to better results. Tables 2 and 3 report the dice coefficient, precision and sensitivity of all methods for all subjects in Optima and UMN datasets, respectively. The bold numbers in each row correspond to the maximum among other numbers. Also, Figs 12 and 13 show the average dice coefficients, sensitivity and precision of 600 images in our local UMN dataset (24 subjects, 25 images per subject), and 196 images in the Optima dataset (4 subjects, 49 images per subject), respectively. In these figures, abbreviations GC, KGC, P.M. and Esm mean graph cut, kernel graph cut, proposed method and method proposed in [31], respectively. In Optima dataset, the proposed method achieves about 5%, 11%, 18%, 25% and 7% higher dice coefficients compared to GC, KGC and method in [31, 52, 53], respectively. Also, the proposed method with sensitivity of 87% outperforms GC, KGC and the method in [31] with sensitivity of 80%, 86% and 66%, respectively. GC has better precision of 90% in comparison with the proposed method with 85%. In local UMN dataset, the proposed method outperforms the GC and KGC, by 12% and 16%, with respect to dice coefficient, 11% and 6% with respect to sensitivity, and 2% and 12% with respect to precision, respectively.

**Table 2:** Average dice coefficients, sensitivity and precision of the proposed method in Optima dataset.

		GC [43]			KGC [44]			Method in [31]			Method in [52]			Method in [53]			Prop. Method		
	Sub	E.1	E. 2	Ave.	E.1	E. 2	Ave.	E.1	E. 2	Ave.	E. 1	E. 2	Ave.	E. 1	E. 2	Ave.	Expr.1	Expr. 2	Ave.
Dice Coeff.	1	73.49	72.96	73.22	80.43	79.10	79.76	71.40	68.17	69.78	61	56	58.5	72	76	74	82.96	82.90	**83.02**
	2	73.90	71.68	72.79	55.10	55.11	55.10	45.49	45.81	45.65	79	76	77.5	84	84	84	78.11	79.09	**77.13**
	3	78.46	82.33	80.40	75.35	79.34	77.35	69.54	65.01	67.27	43	42	42.5	72	75	73.5	82.23	80.36	**84.10**
	4	78.12	77.91	78.01	71.78	71.56	71.67	71.15	72.55	71.85	46	45	45.5	64	67	65.5	80.75	80.87	**80.63**
	Ave.	75.99	76.22	76.10	70.66	71.27	70.97	64.39	62.88	63.63	57.25	54.75	56	73	75.5	74.25	81.01	80.80	**81.22**
Sensitivity	1	70.81	69.95	70.38	82.19	78.56	80.37	72.49	66.75	69.62	NA	NA	NA	NA	NA	NA	84.43	80.94	**82.68**
	2	96.79	92.25	94.52	99.04	94.54	**96.79**	70.45	64.71	67.58	NA	NA	NA	NA	NA	NA	98.94	94.45	96.70
	3	75.72	81.49	78.60	85.13	90.95	**88.04**	47.38	54.84	51.11	NA	NA	NA	NA	NA	NA	85.18	90.75	87.96
	4	78.78	78.54	78.66	80.59	80.22	80.40	77.79	77.56	77.67	NA	NA	NA	NA	NA	NA	84.49	83.70	**84.09**
	Ave.	80.52	80.55	80.54	86.73	86.06	86.40	67.02	65.96	66.49	NA	NA	NA	NA	NA	NA	88.26	87.46	**87.85**
Precision	1	93.00	95.71	**94.36**	85.06	86.55	85.81	54.87	59.61	57.24	NA	NA	NA	NA	NA	NA	84.03	87.53	85.78
	2	74.36	74.45	74.40	54.18	54.14	54.16	51.12	51.34	51.23	NA	NA	NA	NA	NA	NA	78.48	78.58	**78.53**
	3	94.89	96.10	**95.49**	79.88	79.96	79.92	30.93	37.99	34.46	NA	NA	NA	NA	NA	NA	85.45	85.48	85.47
	4	96.97	97.24	**97.11**	88.62	88.99	88.81	54.98	59.50	57.24	NA	NA	NA	NA	NA	NA	93.20	93.58	93.39
	Ave.	89.80	90.87	**90.34**	76.93	77.41	77.17	47.97	52.11	50.04	NA	NA	NA	NA	NA	NA	85.29	86.29	85.79

**Table 3:** Average dice coefficients, sensitivity and precision of the proposed method in our local UMN dataset.

	Dice Cofficients						Sensitivity						Precision					
	GC [43]		KGC [44]		Prop. Method		GC [43]		KGC [44]		Prop. Method		GC [43]		KGC [44]		Prop. Method	
Sub	E.1	E. 2	E.1	E. 2	E.1	E. 2	E.1	E. 2	E.1	E. 2	E.1	E. 2	E.1	E. 2	E.1	E. 2	E.1	E. 2
1	78.68	75.34	79.07	74.76	**85.68**	**81.81**	74.39	71.24	83.03	79.40	**84.38**	**81.01**	**99.59**	**99.43**	89.47	88.97	96.70	95.88
2	77.79	77.83	86.11	82.03	**89.49**	**85.36**	84.24	80.33	92.42	88.31	**94.38**	**90.36**	86.56	86.47	86.22	86.24	**87.80**	**87.57**
3	56.54	53.05	56.56	51.91	**85.81**	**79.40**	50.70	47.26	67.23	62.30	**81.82**	**75.08**	**95.19**	**94.57**	77.98	77.94	97.32	94.54
4	71.94	72.26	69.51	69.49	**82.81**	**82.34**	61.70	62.15	**79.60**	**79.88**	84.92	84.89	**96.67**	**96.56**	73.90	73.90	86.36	85.50
5	45.80	43.70	25.20	24.64	**68.25**	**66.02**	56.10	53.13	54.73	53.18	**67.90**	**64.26**	68.44	68.25	53.98	53.96	**84.66**	**84.60**
6	73.32	68.61	61.34	57.10	**85.91**	**80.53**	81.38	76.73	78.80	74.62	**87.78**	**82.34**	86.93	86.65	76.84	76.45	**89.96**	**89.08**
7	56.71	52.61	46.32	42.00	**67.31**	**63.13**	57.14	54.03	56.70	52.05	**66.37**	**63.28**	**83.63**	**83.31**	79.18	79.15	83.14	82.18
8	**86.89**	86.25	79.06	79.06	86.85	**86.29**	88.43	87.18	86.39	86.14	**92.75**	**91.56**	**90.44**	**90.59**	87.11	87.19	89.29	89.80
9	76.65	72.61	74.95	70.92	**83.00**	**78.81**	79.37	75.32	78.24	74.30	**89.31**	**84.95**	**86.78**	**86.79**	85.00	84.94	85.94	85.94
10	76.54	76.39	76.57	76.44	**84.63**	**80.51**	74.39	70.24	77.86	73.59	**82.91**	**78.44**	**92.55**	**92.64**	89.51	89.62	87.94	88.11
11	72.22	67.74	55.28	51.02	**86.51**	**82.26**	74.29	69.78	77.00	72.63	**82.23**	**77.62**	86.48	86.58	67.16	67.24	**93.37**	**93.71**
12	71.34	68.03	69.53	66.08	**82.42**	**79.43**	62.61	59.60	67.56	64.88	**77.15**	**74.84**	**98.12**	**98.14**	93.25	93.42	97.21	97.43
13	62.61	62.57	64.67	64.56	**89.90**	**89.85**	76.83	76.96	84.06	84.02	**88.31**	**88.13**	74.98	75.06	68.92	69.01	**96.61**	**96.74**
14	63.48	59.26	57.34	53.47	**81.07**	**76.98**	62.24	57.92	65.87	61.82	**78.27**	**73.80**	**94.57**	**94.60**	78.60	78.87	89.49	89.69
15	61.94	53.73	58.19	50.26	**74.16**	**66.07**	64.41	56.20	66.02	57.76	**74.24**	**66.04**	89.64	89.67	81.15	81.30	**88.21**	**88.32**
16	71.17	71.07	69.21	69.20	**87.62**	**83.57**	88.08	84.04	88.63	84.69	**90.34**	**86.29**	79.66	79.62	77.90	77.85	**93.10**	**93.07**
17	73.03	72.17	88.31	83.58	**91.52**	**86.48**	79.57	74.88	**89.29**	**84.13**	90.71	85.36	84.52	84.77	89.12	89.41	**93.38**	**93.43**
18	66.27	58.43	66.93	59.42	**84.70**	**77.06**	59.55	51.68	67.45	59.86	**81.33**	**73.30**	**96.97**	**98.12**	85.12	85.67	93.57	94.37
19	73.93	73.64	43.31	43.26	**86.38**	**82.09**	72.30	67.93	**77.73**	**73.44**	83.24	78.57	**95.27**	**95.48**	58.07	58.45	98.46	98.82
20	65.76	57.41	69.75	63.05	**85.40**	**75.13**	75.35	62.68	87.05	74.09	**88.61**	**75.60**	77.52	78.22	68.67	69.85	**86.05**	**88.32**
21	81.11	70.62	76.04	66.16	**89.00**	**78.38**	77.60	64.02	84.37	69.73	**85.61**	**69.97**	**95.30**	**95.04**	80.93	80.61	94.57	94.23
22	71.33	64.40	61.50	55.00	**77.16**	**70.19**	83.81	73.53	**90.66**	**78.71**	87.94	76.80	76.00	75.97	60.03	59.89	**83.20**	**83.16**
23	87.83	79.47	90.85	82.49	**91.68**	**82.66**	85.90	73.21	**94.29**	**79.51**	87.77	72.97	91.42	91.27	88.26	88.12	**96.43**	**96.20**
24	81.37	77.12	87.27	83.22	**88.79**	**84.77**	74.72	70.33	86.72	82.41	**86.85**	**82.57**	**94.49**	**94.49**	89.56	89.73	91.93	92.19
Ave.	71.01	67.26	67.20	63.30	**84.00**	**79.13**	72.71	67.52	78.40	72.98	**83.96**	**78.25**	88.41	88.43	78.58	78.66	**91.03**	**90.95**
Ave.	69.13		65.25		**81.56**		70.11		75.69		**81.10**		88.42		78.62		**90.99**	

### 3D fluid segmentation results

**Fig 14:**
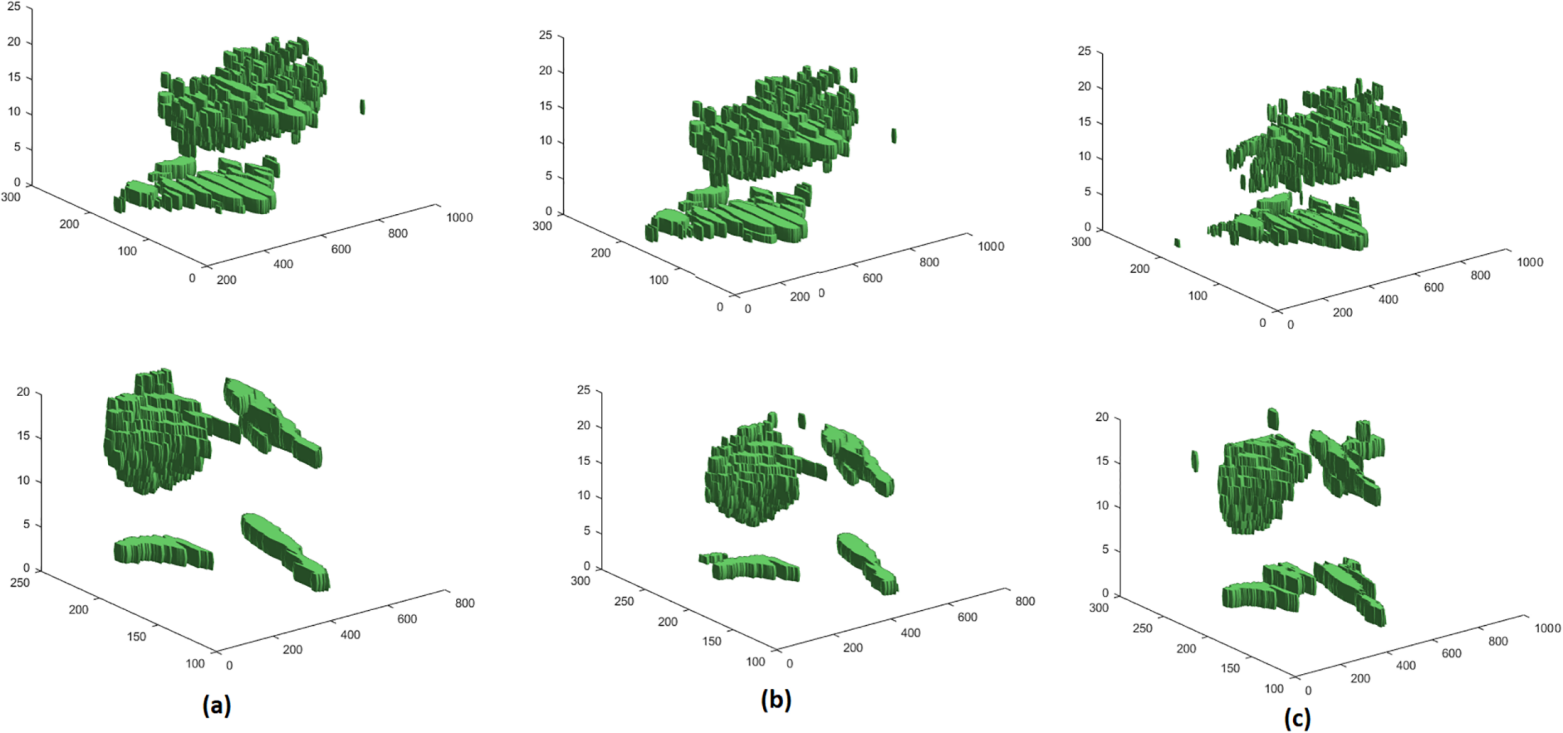
Segmented 3D fluids for two OCT volumes in two rows. Volume segmented by (a): expert 1, (b): expert 2 and (c): proposed method.

**Fig 15:**
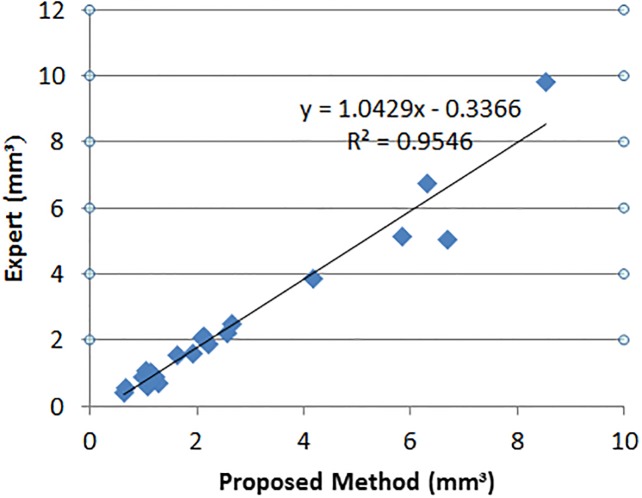
Linear regression analysis between the volume of fluid segmented by the proposed method and expert.

In 3D OCT volumes, if there are fluid regions in consecutive 2D scans, fluid volumes are obtained. In 21 OCT volumes (out of 24 volumes) in local UMN dataset there are fluid regions in subsequence scans; therefore, 3D fluid volumes can be segmented. Fig 14 shows the segmented 3D fluids for two OCT volumes. Fig 15 depicts the linear regression analysis between the volume of fluid segmented by the proposed method and expert (intersection between experts 1 and 2). For 3D fluid segmentation, the proposed method has been compared and reported with methods in [43, 44] in Table 4.

**Table 4:** Mean and standard deviation of TPR and FPR in 3D fluid segmentation achieved by the proposed method and other methods.

Method	# of Subjects	TPR(%)	FPR(%)
GC [43]	21	70.63±14.37	1.47±1.82
KGC [44]	21	82.49± 11.35	0.68± 0.53
Proposed method	21	**90.17 ± 3.74**	**0.74±1.51**

### Seed initialization robustness

Graph cut is very sensitive to seed points. Changes in seed points lead to significant changes in segmentation results (see Fig 6). In the proposed seed initialization method in Algorithm 3, seed points are selected randomly in specific clusters in NS domain in steps 9-10. Therefore, in different runs of the Algorithm 3, different seed initializations are obtained. To show how fluid segmentation results are affected from different seed initializations, 100 scans are selected randomly from each dataset and then 10 seed point sets are obtained from 10 different runs of Algorithm 3. Table 5 reports the average and standard deviation of dice coefficient, sensitivity and precision. Also, the accuracy of fluid and tissue seed points are reported in this Table. It can be concluded that different seed initializations obtained from Algorithm 3 lead to minor changes (low standard deviation) in fluid segmentation results.

**Table 5:** Seed initialization robustness analysis.

	FG Acc	BG Acc	Dice Coff.	Sensitivity	Precision
UMN	88.81±0.42	96.63±0.12	81.59±1.41	98.14±0.2	69.87±1.90
Optima	82.34±1.58	94.67±0.32	61.82±2.98	77.74±1.32	56.41±3.22

### Inter-observer varibility

In this study, correlation between observers referred as grader 1 (G1) and grader 2 (G2) is analyzed and reported in Table 6. When the observer correlation is high, the segmentation errors are due to the segmentation algorithm, not inter-observer variation. The observer correlation of the used datasets in this study are 92.88% and 91.15% with respect to the dice coefficients in the UMN and Optima datasets, respectively. From the low inter-observer variabilities in both datasets, it can be concluded that the segmentation errors of the proposed algorithm stem from the different steps of the algorithm, not inter-observer variability.

**Table 6:** Inter-observer variability analysis.

		Average of Automatic vs. G1 and G2	Inter-Observer	Automatic vs.G1 and G2 Intersection
UMN dataset	Dice Coff.	78.97	92.88%	81.21%
	Sensitivity	77.9	92.69%	81.04%
	Precision	90.49	97.10%	89.79%
Optima dataset	Dice Coff.	81.22%	91.15%	81.48%
	Sensitivity	87.85%	91.39%	91.02%
	Precision	85.79%	94.35%	83.97%

### Clusters for fluid and tissue seed sets

One of the challenges of the automated seed initialization method in Algorithm 3 is to determine the clusters for fluid and tissue seed selection. Based on the transformation method in Algorithm 1, the fluid regions have the maximum intensity. After sorting in step 8 of Algorithm 3, the first cluster (with the biggest center value) is the best candidate for fluid seed selection. The challenge is that in some scans with very dark background, this cluster contains only background regions. In such cases, fluid regions are assigned to second or maybe third cluster. Therefore, fluid seed selection from the first cluster lead to incorrect seeds and low accuracy segmentation. To address this problem, fluid seed points are selected from the first and second clusters. Table 5 reports the accuracy of fluid (foreground) and tissue (background) seed points selection.

### OCT scans without fluid

For normal scans (without fluid), a few fluid seed points are selected in step 9 of Algorithm 3. This leads to small segmented fluid regions in graph cut segmentation and these regions are ignored in step 6 of Algorithm 4. Therefore, OCT scans without fluid are detected as normal scans automatically.

### Cirrus OCT data

To evaluate the performance of the proposed method on OCT data acquired from other imaging devices, a Cirrus OCT dataset from Optima challenge 2015 including 512 Bscans from 4 subjects is used. Table 7 reports dice coefficients of the proposed method and methods proposed by first and second ranks in this challenge. The method in third rank has not been applied to Cirrus data. The proposed method achieved the dice coefficient of 78% which outperforms methods in first and second rank with 64% and 44%, respectively. It may be noted that the proposed method has a better performance on Spectralis datasests which is due to higher quality of these datasets.

**Table 7:** Average dice coefficients of the proposed method in Cirrus dataset.

	Method in [52]			Method in [53]			Proposed Method		
Sub	E. 1	E. 2	Ave.	E. 1	E. 2	Ave.	E. 1	E. 2	Ave.
1	0.41	0.40	0.405	0.69	0.71	0.70	0.8378	0.8345	0.8361
2	0.47	0.45	0.46	0.85	0.84	0.845	0.9194	0.9042	0.9118
3	0.50	0.50	0.50	0.64	0.61	0.625	0.6727	0.6680	0.6703
4	0.41	0.41	0.41	0.42	0.38	0.40	0.7235	0.7080	0.7157
Ave.	**0.4475**	**0.44**	**0.4437**	**0.65**	**0.63.5**	**64.25**	**0.7883**	**0.7786**	**0.7834**

### Fluid types

**Fig 16:**
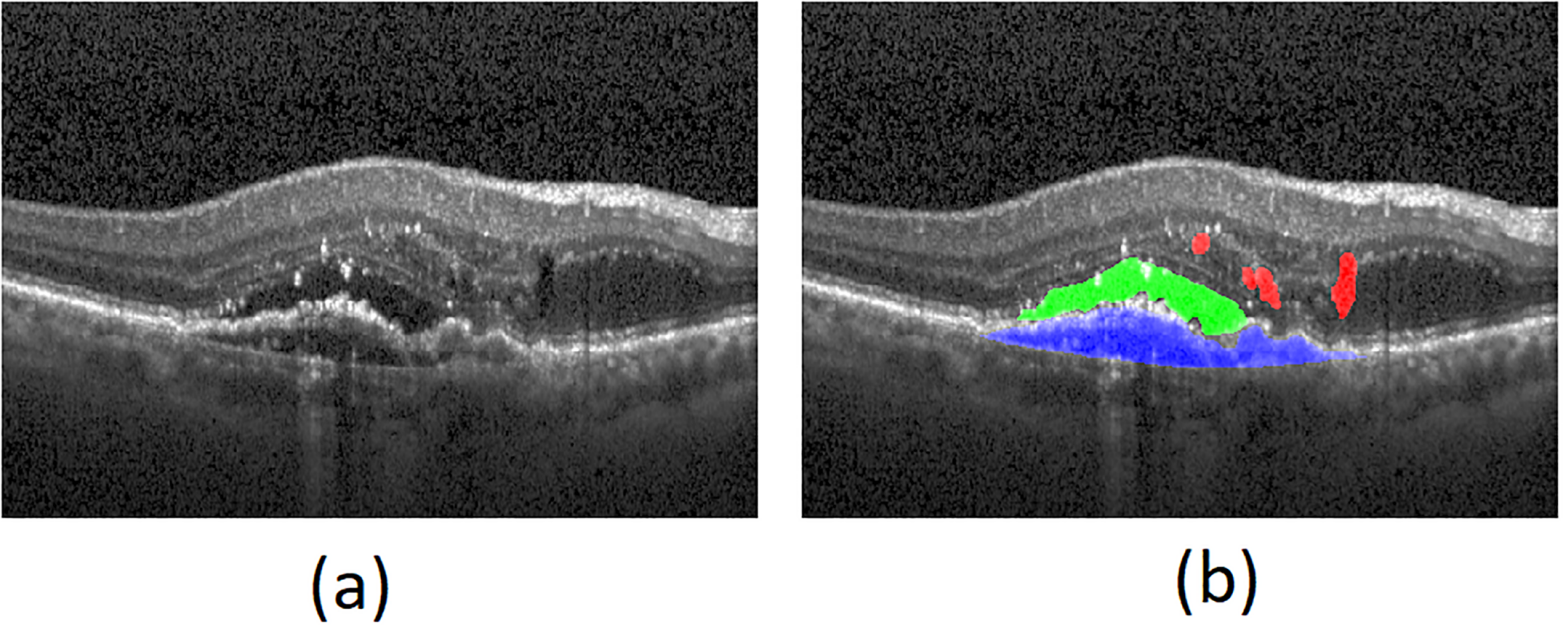
Intraretinal, subretinal and sub-RPE fluid types. (a) Input OCT scan (b) intraretinal, subretinal and sub-RPE fluids in red, green and blue colors, respectively.

From OCT Bscans, three types of fluid including intraretinal, subretinal and sub-RPE are clinically distinguishable. Intraretinal fluid are revealed as contiguous fluid-filled spaces containing columns of tissue. Some times, these spaces appear as separated hyporeflective cystoid pockets. When these regions are too small, they are referred as micro-cyst regions. Accumulation of a clear or lipid-rich exudate underneath retinal space creates subretinal fluid. Note that intraretinal and subretunal fluid types are common in both AMD and DME subjects. In early and neovascular AMD, detachment of the RPE layer along with the overlying retina from the remaining Bruch’s membrane occurs which is referred as Pigment Epithelial Detachment (PED). This is due to the accumulation of sub-RPE fluid. This type of abnormality appears as serous, fibrovascular and drusenoid. Fig 16 represents intraretinal, subretinal and sub-RPE fluid with red, green and blue colors, respectively [6].

In this research, the collected UMN dataset contains all three fluid types while datasets from Optima challenge contain only intraretinal fluid. Intraretinal and subretinal fluids are segmented by Algorithms 1, 3 and 4. Sub-RPE fluid is segmented by the proposed RPE segmentation and RPE flattening (Algorithm 2). It may be worth mentioning that, the proposed methods for sub-RPE fluid segmentation is not affected by the intensity corresponding to fluid regions which leads to the segmentation of any type of RPE elevation.

## Conclusion

In line with advanced EAMD treatment methods, OCT has emerged as an essential adjunct for the diagnosis and monitoring of this disease and the ability to accurately segment fluid as an EAMD biomarker is a prerequisite for ophthalmologists in treatment process. In this research, an automated algorithm for 3D fluid volume segmentation in subjects with EAMD pathology has been proposed based on graph cut, graph shortest path and neutrosophic sets. To show the efficiency of the proposed method, it was tested on two OCT datasets. Results show that fluid volumes obtained from our proposed algorithm closely correlated to the manual segmentations of the two ophthalmologist experts in 3D fluid volume and achieve better performance in comparison with prior methods with respect to dice coefficient, sensitivity and precision measures in 2D individual scans. Future efforts will be directed towards fine-tuning the algorithm for OCT images obtained from other manufacturers and DME subjects which do not have RPE elevation. Finally, reproducibility studies between segmentation following repeat imaging can be addressed as another future work.
